# Peptide OH‐CATH30 Mitigates Cachexia‐Induced Muscle Atrophy via Modulation of TLR4‐Associated Inflammation

**DOI:** 10.1002/jcsm.70195

**Published:** 2026-01-29

**Authors:** Qiquan Wang, Jian Li, Mengqi Yang, Caifen Guo, Ming Zhang, Chunping Huang, Xiang Wang, Dongqin Zhang, Lin Zeng, Hao Ke, Yunling Wen, Shengan Li, Wenhui Lee, Limin Zhao, Xinqiang Lan, Yang Xiang

**Affiliations:** ^1^ Metabolic Control and Aging, Human Aging Research Institute and School of Life Science Nanchang University and Jiangxi Key Laboratory of Aging and Diseases Nanchang China; ^2^ Department of Sports Medicine The Beijing Jishuitan Hospital Guizhou Hospital Guiyang China; ^3^ Department of Urology The Affiliated Hospital of Guizhou Medical University Guiyang China; ^4^ Department of Radiation Therapy Yunnan Cancer Hospital/Third Affiliated Hospital of KunMing Medical University Kunming China; ^5^ HeartSeek Biotechnology Kunming China; ^6^ Institutional Center for Shared Technologies and Facilities of the Kunming Institute of Zoology Chinese Academy of Sciences Kunming China; ^7^ Department of Gastroenterology The First Affiliated Hospital of Kunming Medical University Kunming China; ^8^ Department of Pathogen Biology and Immunology, Faculty of Basic Medical Science Kunming Medical University Kunming Yunnan China; ^9^ Key Laboratory of Animal Models and Human Disease Mechanisms of the Chinese Academy of Sciences, Key Laboratory of Bioactive Peptides of Yunnan Province Kunming Institute of Zoology, The Chinese Academy of Sciences Kunming China

**Keywords:** cachexia, inflammation, muscle atrophy, OH‐CATCH30, protein degradation, TLR4

## Abstract

**Background:**

Cachexia, characterized by severe weight loss and muscle atrophy, frequently occurs in chronic conditions such as sepsis, cancer and chemotherapy, with limited effective treatments. Despite similar clinical manifestations, the underlying mechanisms across different disease contexts remain unclear. Identifying common pathways could lead to novel therapies. This study examines the role of Toll‐like receptor 4 (TLR4), which is upregulated in various cachexia models, and assesses the therapeutic potential of the TLR4‐inhibiting peptide OH‐CATH30 in mitigating muscle atrophy.

**Methods:**

In vivo models using 8‐week‐old mice treated with lipopolysaccharide (LPS), 4T1 tumour cells and cisplatin were used to investigate common pathways in cachexia. In vitro models were established by treating C2C12 myotubes with TNF‐α, 4T1 culture supernatants and cisplatin. OH‐CATH30's effects on muscle atrophy were assessed by measuring myotube diameter, grip strength, muscle weight and muscle fibre cross‐sectional area (CSA) via H&E staining. RNA‐seq, qPCR, ELISA and Western blotting were performed to explore pathways in cachexia‐induced muscle atrophy and OH‐CATH30's action mechanism.

**Results:**

Transcriptomic analysis showed significant enrichment of inflammation and protein degradation pathways in skeletal muscle in LPS‐induced sepsis, 4T1 tumour‐induced cancer cachexia and cisplatin‐induced cachexia models, with upregulated expression of TLR4 pathway genes such as *Cd14*, *Tlr4* and *Irak4* (*p* < 0.05). In myotube atrophy models induced by TNF‐α, 4T1 and cisplatin, OH‐CATH30 significantly increased MyHC protein levels (*p* < 0.05) and myotube diameter (*p* < 0.05). In mouse cachexia models induced by LPS, 4T1 and cisplatin, OH‐CATH30 treatment significantly increased body weight (*p* < 0.05), muscle weight (*p* < 0.001), CSA (*p* < 0.05) and improved grip strength (*p* < 0.05). Transcriptomic analysis further revealed that OH‐CATH30 treatment downregulated expression of inflammation and protein degradation‐related genes across all cachexia models. In 4T1‐treated mice, qPCR confirmed OH‐CATH30 reduced mRNA levels of *Il6* (*p* = 0.05), *Mstn* (*p* < 0.0001) and protein degradation genes such as *Trim63*, *Fbxo32*, *Bnip3*, *Gabarapl1* and *Ulk1* (*p* < 0.05). ELISA showed reduced serum IL‐6 levels, and Western blot confirmed downregulation of atrogin1 (*p* < 0.05) and autophagy marker LC3II (*p* < 0.05) with OH‐CATH30 treatment. Pharmacological inhibition of TLR4 using TAK‐242 recapitulated the protective effects of OH‐CATH30, with no additive benefit observed (*p* > 0.05).

**Conclusions:**

Our findings underscore the critical role of TLR4 signalling in cachexia‐associated muscle wasting across different disease contexts and demonstrate the efficacy of OH‐CATH30, a TLR4 inhibitor, in alleviating muscle atrophy in various cachexia models.

## 1 Introduction

Cachexia is a complex, multifactorial syndrome marked by severe weight loss, primarily due to skeletal muscle atrophy, with or without fat reduction [1]. This syndrome is commonly observed in patients with chronic diseases such as sepsis [2], cancer [1] or those undergoing chemotherapy, particularly with agents like cisplatin [3]. Cachexia significantly burdens healthcare systems due to its high morbidity and mortality rates, impacting patients' quality of life, functional status and overall prognosis [4]. In cancer patients, cachexia is especially detrimental, as it worsens treatment outcomes and decreases survival rates [1]. Despite these serious consequences, effective treatments for cachexia‐related muscle waste remain lacking, underscoring the urgent need for further research into its mechanisms and potential therapies.

Inflammation‐induced protein degradation is a key driver of muscle atrophy in cachexia [1]. Chronic inflammation, a common feature of sepsis, cancer cachexia and chemotherapy toxicity [2, 3, 5], activates catabolic pathways in skeletal muscle, leading to protein breakdown and muscle loss [1, 6, 7]. Elevated pro‐inflammatory cytokines, such as TNF‐α and IL‐6, bind to muscle cell receptors, triggering intracellular signalling cascades that upregulate the ubiquitin‐proteasome and autophagy‐lysosome systems—both of which degrade muscle proteins [8, 9]. Although inflammation's role in muscle atrophy is well documented, the specific inflammatory pathways involved across different cachectic conditions are not fully understood.

Toll‐like receptors (TLRs), particularly TLR4, are central to inflammatory signalling in various tissues, including skeletal muscle [10]. Studies show that TLR4 expression is significantly elevated in the skeletal muscle of cachectic patients and animal models [11, 12], and TLR4 deletion can protect against muscle wasting in conditions like Lewis lung carcinoma (LLC) [13]. Thus, targeting TLR4 signalling could be a promising therapeutic strategy to prevent cachexia‐associated muscle atrophy.

Bioactive peptides, short sequences of amino acids, have shown significant potential in regulating pathways related to muscle protein synthesis, degradation, and inflammation [14]. Compared to conventional therapies, bioactive peptides often offer high specificity and minimal off‐target effects [15], making them well‐suited for chronic conditions like cachexia. For instance, the myostatin pro‐peptide inhibits myostatin, showing potential in treating muscle‐wasting diseases like cachexia and sarcopenia [16, 17, 18], while GLP‐1, known for its role in glucose metabolism, may help address muscle wasting in obesity [19] and chronic kidney disease [20]. Exploring such peptides offers promising therapeutic avenues for cachexia.

In this study, we performed transcriptomic analyses to uncover molecular changes driving muscle atrophy induced by sepsis, cancer and chemotherapy. Inflammatory and catabolic pathways, particularly TLR4 signalling, were prominently enriched. We further evaluated OH‐CATH30, a natural TLR4 inhibitory peptide [21], and found it effectively mitigated muscle wasting and reversed inflammatory markers across all three cachexia models. These findings support targeting TLR4 with OH‐CATH30 as a promising therapeutic strategy for cachexia.

## 2 Methods

### 2.1 Cell Culture and OH‐CATH30 Treatment

The C2C12 murine myoblast was obtained from Shanghai Cell Bank, Chinese Academy of Sciences, and maintained in growth medium (high‐glucose DMEM with 10% FBS) at 37°C with 5% CO_2_. To study the effects of OH‐CATH30 on TNF‐α and cisplatin‐induced myotube atrophy, cells were cultured in differentiation medium (DMEM with 2% horse serum) for 6 days to form myotubes. Subsequently, differentiated myotubes were treated with 10‐ng/mL TNF‐α or 150‐μM cisplatin and OH‐CATH30 (0, 2.5, 5 μg/mL) for 48 h.

### 2.2 Animal Model and OH‐CATH30 Treatment

Eight‐week‐old C57BL/6 and BALB/c mice were obtained from Changsha Tianqin Biotechnology, with protocols approved by the Nanchang University Institutional Animal Care and Use Committee (NCULAE‐20221130020). For the LPS‐induced muscle atrophy model, C57BL/6 mice were divided into control, LPS‐treated, and LPS + OH‐CATH30 groups (6–8 mice per group). Mice received LPS (2 mg/kg/day, ip), followed by daily injections of OH‐CATH30 (5 mg/kg/day) or PBS for 10 days. In the 4T1‐induced cachexia model, BALB/c mice were divided into control and cachexia groups (20 mice per group). Following subcutaneous injection of 4T1 cells (1 × 10^6^) 1 week, cachexia mice were treated with PBS or OH‐CATH30 (5 mg/kg, ip, every 3 days) for 24 days. In the cisplatin‐induced muscle atrophy model, C57BL/6 mice were assigned to control, cisplatin‐treated and cisplatin + OH‐CATH30 groups (10 mice per group). After cisplatin injections (3 mg/kg/day, ip), mice received daily OH‐CATH30 or PBS for 8 days. Body weight and food intake were monitored daily across all three models. After each experiment, mice were anaesthetised, and the muscles were collected, weighed, frozen for analysis or fixed in paraformaldehyde for histology.

In addition, other experiments including cell‐based experiments and functional tests were also encompassed in this research. For more details, please refer to Supporting Information.

### 2.3 RNA Isolation and Quantitative RT‐PCR

Cell or muscle samples were treated with TRIzol reagent and homogenized with a tissue lyser. RNA extraction followed the manufacturer's protocol. One microgram of RNA was reverse‐transcribed into cDNA using the PrimeScript RT reagent Kit with gDNA Eraser. Quantitative PCR (qPCR) was conducted on a Real‐time qPCR Detection System using SYBR Green PCR Master Mix, with data analysed via the 2^‐ΔΔCt^ method. Primer sequences are listed in Table S1.

### 2.4 Western Blot Analysis

Protein samples were separated by SDS‐PAGE and transferred to a PVDF membrane, then blocked with 3% BSA in PBS with 0.1% Tween20 for 1 h at room temperature. After blocking, membranes were incubated overnight at 4°C with primary antibodies. The next day, membranes were incubated with secondary antibodies for 1 h at room temperature. Protein bands were visualized with Super Signal chemiluminescence reagents and quantified using ImageJ software. Control samples were set to 100% for bar graph representation, and percentage changes in intensity were calculated accordingly, as described previously [22]. Please refer to the Supporting Information for the antibodies used in the Western blot analysis.

### 2.5 Statistical Analysis

All experimental values are expressed as mean ± SD. All data were analysed using Prism v 8.0 software. Two‐sample comparisons were performed using unpaired Student's *t* test. Multiple comparisons were performed using one‐way analysis of variance (ANOVA), with post hoc contrasts by Dunnett's multiple‐comparison test; *p* < 0.05 were considered statistically significant.

## 3 Results

### 3.1 Muscle Atrophy Across Cachexia Models Is Associated With Increased Inflammation

**FIGURE 1:**
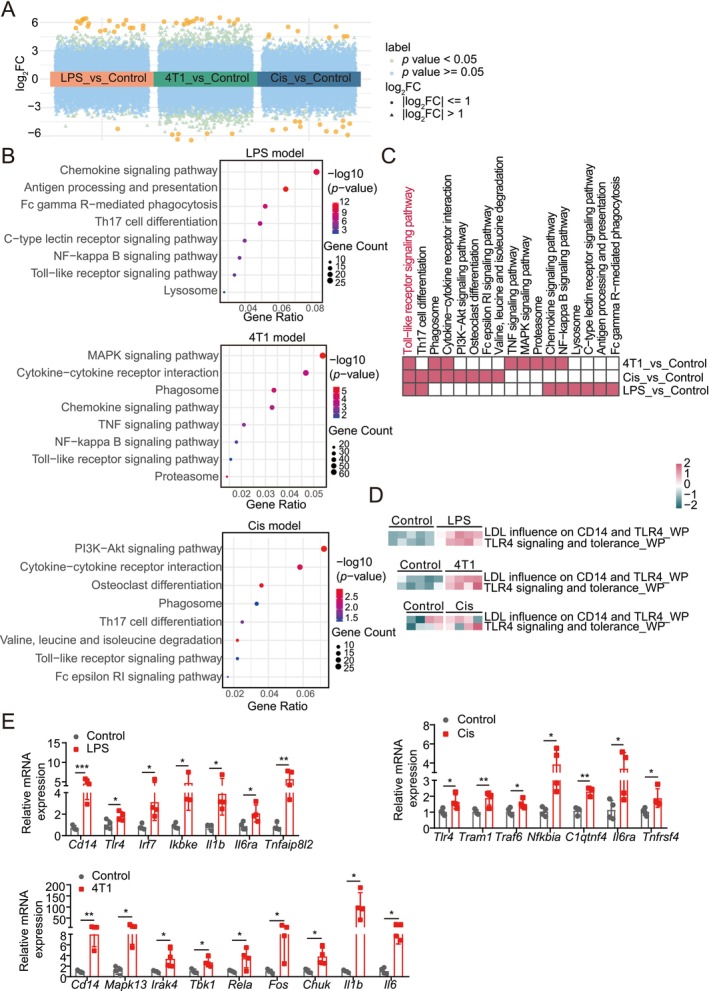
Transcriptomic analysis of distinct cachexia models. (A) Volcano plots visualizing the differential expression analysis results of the transcriptomic data from the gastrocnemius (GA) muscle in control and model groups for three cachexia models: lipopolysaccharide (LPS)‐induced sepsis, 4T1 tumour‐induced cancer cachexia and cisplatin (Cis)‐induced chemotherapy‐associated cachexia. Significant upregulated and downregulated genes are identified with *p* < 0.05 and |log2FC| > 1. Orange dots represent the top 20 genes with the highest |log_2_ fold change| in each comparison group. (B) Kyoto Encyclopedia of Genes and Genomes (KEGG) pathway enrichment dendrogram for differentially expressed genes (DEGs) between the model and control groups across all three cachexia models, showing the top 8 significantly enriched pathways (*p* < 0.05) per model. (C) Comparative analysis of enriched pathways identified in the transcriptomic data across the three cachexia models: LPS‐induced sepsis, 4T1 tumour‐induced cancer cachexia and cisplatin‐induced chemotherapy‐associated cachexia. (D) Single‐sample gene set enrichment analysis (ssGSEA) of TLR4‐related pathways across three cachexia models. (E) qPCR analysis of TLR4 signalling‐related gene expression in the three cachexia models. Values are mean ± SD. Significance determined using unpaired *t* test. *p < 0.05, **p < 0.01 and ***p < 0.001 versus control group.

To examine the mechanisms of skeletal muscle atrophy in cachexia, we generated three mouse cachexia models: LPS‐induced sepsis, 4T1 tumour‐induced cancer cachexia and cisplatin (Cis)‐induced chemotherapy‐associated cachexia (Figure S1A). All models showed significant reductions in body weight and muscle mass, specifically in the GA, TA and SOL muscles (Figure S1B), confirming successful cachexia induction. We conducted transcriptomic analysis of GA muscle tissue across models to uncover molecular alterations in muscle wasting. Principal component analysis (PCA) showed a clear separation between control and model groups along principal components (PC1 and PC2), reflecting substantial transcriptional differences (Figure S2). Differential gene expression analysis identified numerous genes that were significantly upregulated or downregulated in model groups compared to controls, as shown in the volcano plots (Figure 1A and Table S2).

Kyoto Encyclopedia of Genes and Genomes (KEGG) pathway enrichment analysis of these differentially expressed genes (DEGs) highlighted significant upregulation of inflammation‐related pathways, including the NF‐κB signalling pathway and cytokine‐cytokine receptor interactions, along with protein degradation pathways, such as the proteasome and autophagy‐lysosome pathways (Figure 1B). Protein–protein interaction (PPI) network analysis demonstrated a consistent interaction network across models, with core inflammatory‐related genes, such as *Syk*, *Fcgr4*, *CD86*, *Ccr5* and *Kit*, forming central nodes (Figure S3A). Further transcriptomic analyses confirmed upregulation of these core genes in cachexia models compared to controls (Figure S3B and Table S3). Overall, these findings suggest that inflammation pathways are closely associated with muscle wasting in cachexia, irrespective of the disease model.

### 3.2 TLR4 Signalling Is Upregulated Across Cachexia Models

To clarify the role of inflammation‐related signalling in cachexia, we analysed transcriptomic data and found a significant enrichment of the Toll‐like receptor (TLR) signalling pathway in all cachexia models (Figure 1C), underscoring its potential involvement in cachexia‐induced muscle atrophy. Given TLR4's established role in inflammation and cachexia, we focused on TLR4 signalling activation within each model. Single‐sample gene set enrichment analysis (ssGSEA) indicated robust activation of TLR4‐related pathways in the cachexia models compared to controls (Figure 1D). Several downstream inflammatory genes, including *Chuk*, *Il1b* and *C1qtnf4*, were found to be upregulated in two out of the three cachexia models, suggesting their involvement in shared inflammatory responses (Figures S4–S6 and Table S4). These transcriptomic findings were further supported by qPCR validation, which confirmed consistent upregulation of *Tlr4* and key downstream inflammatory mediators in the three models (Figure 1E). These results suggest that TLR4 serves as a pivotal mediator of inflammation‐driven muscle atrophy in cachexia, positioning it as a potential therapeutic target for preventing muscle wasting across various cachexia conditions.

### 3.3 OH‐CATH30 as a TLR4 Inhibitor Prevents Cachexia‐Induced Myotube Atrophy In Vitro

**FIGURE 2:**
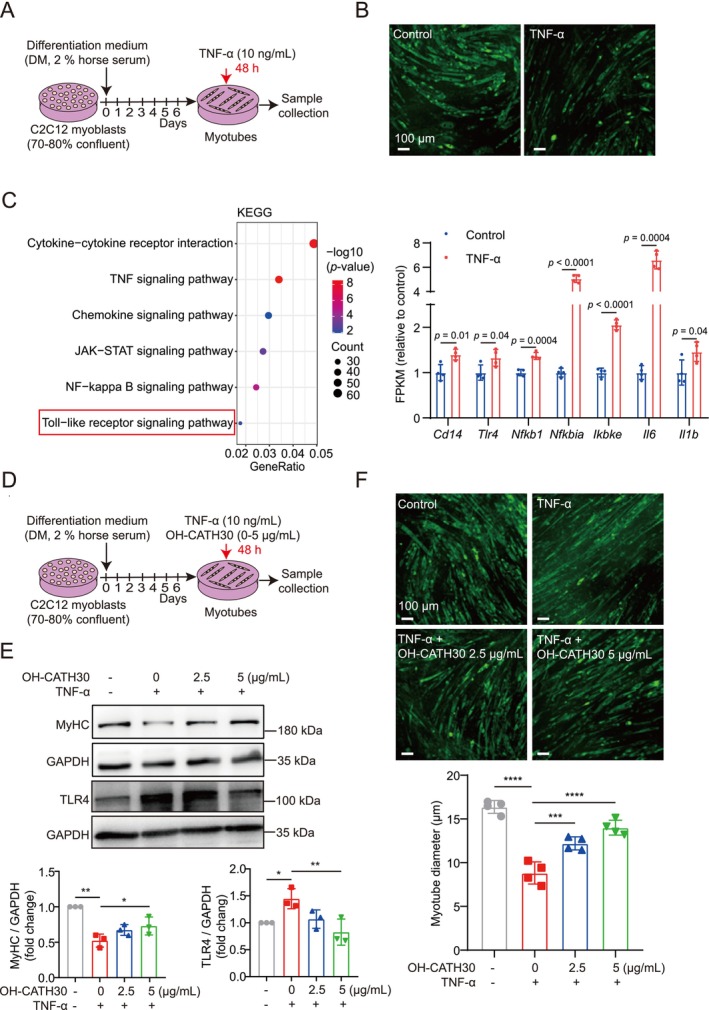
Effects of OH‐CATH30 on TNF‐α treated C2C12 myotubes. (A) Schematic diagram of the experimental design of C2C12 myotubes treated with TNF‐α for 48 h. (B) Immunostaining visualization of differentiated C2C12 myotubes treated with TNF‐α for 48 h. Scale bar: 100 μm. (C) KEGG pathway enrichment dendrogram for differentially expressed genes (DEGs) between TNF‐α‐treated and control C2C12 myotubes (left), alongside a bar chart of TLR4 signalling‐related gene expression in these myotubes (right). (D) Schematic diagram of the experimental design of C2C12 myotubes treated with TNF‐α and OH‐CATH30 together for 48 h. (E) Western blot analysis of MyHC and TLR4 protein expression in C2C12 myotubes co‐treated with TNF‐α (10 ng/mL) and OH‐CATH30 (0–5 μg/mL) for 48 h, with quantification performed using ImageJ. *n* = 3. (F) Immunostaining visualization and quantification of myotube structure in C2C12 myotubes treated with TNF‐α (10 ng/mL) and OH‐CATH30 (0–5 μg/mL). Scale bar: 100 μm. *n* = 4. Values are mean ± SD. Significance determined using one‐way ANOVA (E, F). *p < 0.05, **p < 0.01 and ****p < 0.0001 versus vehicle‐treated group (0 μg/mL OH‐CATH30).

Our prior studies have shown that OH‐CATH30, a 30‐amino acid linear cationic peptide (Figure S7), effectively suppresses the production of pro‐inflammatory cytokines (e.g., IL‐6, TNF‐α and NO) by specifically inhibiting TLR4 in a sepsis model [21]. To investigate the potential of OH‐CATH30 as a TLR4 inhibitor in preventing cachexia‐induced muscle atrophy, we established an in vitro model using C2C12 murine myotubes. Differentiated myotubes were treated with TNF‐α to simulate cachexia‐induced atrophy, which resulted in a significant reduction in myotube diameter (Figure 2A,B), confirming the induction of muscle wasting. Consistent with findings from our in vivo models, transcriptomic analysis (Figure S8 and Table S5) indicated that TNF‐α treatment activated TLR4 signalling and upregulated downstream inflammatory genes in C2C12 myotubes (Figure 2C and Table S6).

We then treated TNF‐α‐exposed C2C12 myotubes with increasing concentrations of OH‐CATH30 (0, 2.5 and 5 μg/mL) and assessed its protective effects (Figure 2D). As anticipated, OH‐CATH30 dose‐dependently attenuated the TNF‐α–induced upregulation of TLR4 protein expression (Figure 2E). Both Western blot and immunofluorescence analyses revealed that OH‐CATH30 treatment significantly attenuated TNF‐α‐induced myotube atrophy in a dose‐dependent manner, as shown by increased MyHC protein level and preserved myotube structure (Figure 2E,F). Furthermore, similar protective effects were observed when myotubes were exposed to conditioned media from 4T1 tumour cells or cisplatin, representing cachexia models associated with cancer and chemotherapy (Figure S9). These results demonstrate that OH‐CATH30, as a TLR4 inhibitor, effectively prevents cachexia‐induced myotube atrophy in vitro.

### 3.4 OH‐CATH30 Attenuates LPS‐Induced Muscle Wasting In Vivo

**FIGURE 3:**
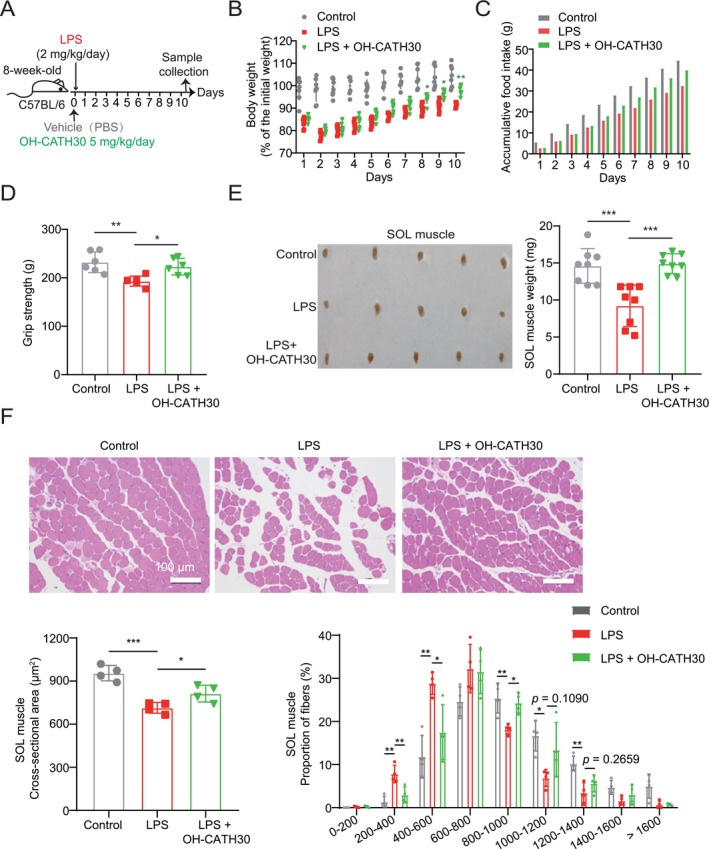
Effects of OH‐CATH30 on LPS‐challenged mice. (A) Schematic diagram of the experimental design: 8‐week‐old male C57BL/6 mice were intraperitoneally injected with LPS (2 mg/kg/day) for 10 days, followed by daily intraperitoneal injections of OH‐CATH30 (5 mg/kg) or PBS. Body weight and food intake were recorded daily. *n* = 6–8 per group. (B) Body weight of each group mice. (C) Average accumulative food intake of each group mice. (D) Grip strength of each group mice. (E) Representative images and weights of SOL muscles for each group of mice. (F) Representative photomicrographs of H&E‐stained soleus muscle sections and cross‐sectional area (CSA) from each group mice. Scale bar: 100 μm. *n* = 4. Values are mean ± SD. Significance determined using one‐way ANOVA (B, D, E and F). *p < 0.05, **p < 0.01 and ***p < 0.001 versus LPS‐treated group.

To evaluate the efficacy of OH‐CATH30 in alleviating cachexia‐related muscle atrophy in vivo, we used an LPS‐induced septic cachexia model. Eight‐week‐old male C57BL/6 mice were treated with LPS to induce septic cachexia and administered OH‐CATH30 or a vehicle control daily for 10 days (Figure 3A). OH‐CATH30 treatment significantly mitigated weight loss on Days 8, 9 and 10 during the body weight recovery phase (Figure 3B). LPS treatment also reduced food intake, and OH‐CATH30 partially restored consumption levels 1 week after LPS administration (Figure 3C), suggesting its potential to reduce cachexia‐induced metabolic dysfunction.

We next assessed the effects of OH‐CATH30 on muscle function and atrophy. Grip strength measurements revealed a significant reduction in strength in LPS‐treated mice, whereas OH‐CATH30‐treated mice showed significantly higher grip strength compared to the LPS‐only group (Figure 3D). Skeletal muscle analysis revealed a significant reduction in the SOL muscle mass following LPS treatment, which was partially preserved in the OH‐CATH30‐treated mice (Figure 3E). Furthermore, histological analysis of muscle fibres showed that LPS‐induced reductions in the cross‐sectional area (CSA) of SOL muscle fibres were significantly improved by OH‐CATH30 treatment (Figure 3F). These findings demonstrate that OH‐CATH30 effectively alleviates muscle atrophy and improves physical function in the LPS‐induced septic cachexia model.

### 3.5 OH‐CATH30 Ameliorates Muscle Wasting in 4T1 Tumour‐Bearing Mice

**FIGURE 4:**
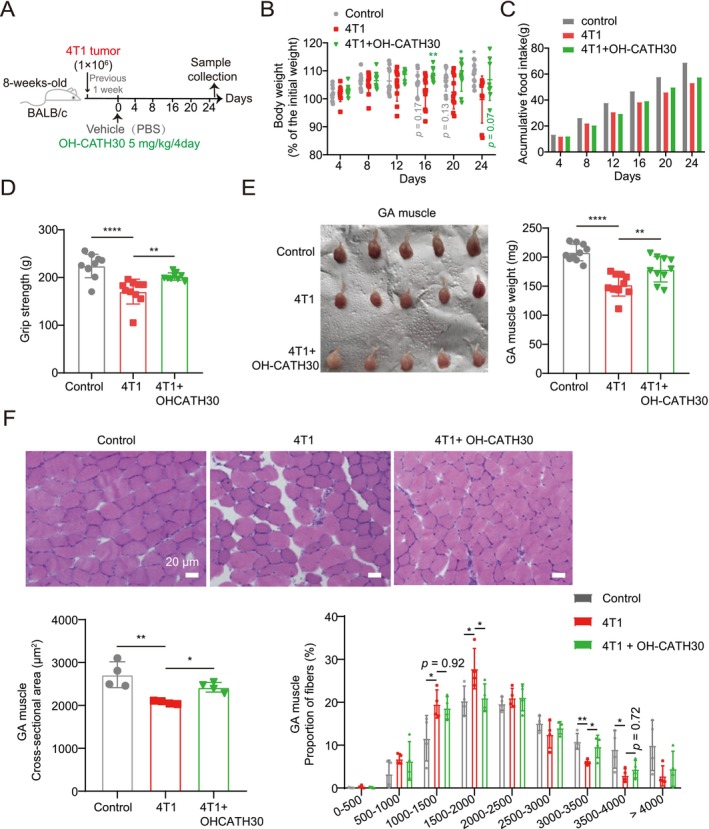
Effects of OH‐CATH30 on 4T1‐tumour mice. (A) Schematic diagram of the experimental design: 8‐week‐old female BALB/c mice were subcutaneously inoculated with 1 × 10^6^ 4T1 breast cancer cells. Starting on day 7 post‐inoculation, mice were treated with OH‐CATH30 (5 mg/kg, ip, every 3 days) or PBS. Body weight and food intake were measured daily. *n* = 10 per group. (B) Body weight of each group mice. (C) Average accumulative food intake of each group mice. (D) Grip strength of each group mice. (E) Representative images and weights of GA muscles for each group of mice. (F) Representative photomicrographs of H&E‐stained gastrocnemius muscle sections and cross‐sectional area (CSA) from each group mice. Scale bar: 20 μm. *n* = 4. Values are mean ± SD. Significance determined using one‐way ANOVA (B, D, E and F). *p < 0.05, **p < 0.01 and ****p < 0.0001 versus 4T1‐tumour group.

To evaluate the efficacy of OH‐CATH30 in a tumour cachexia model, we treated Balb/c mice bearing 4T1 tumours with intraperitoneal injections of OH‐CATH30 every 3 days, starting 1 week after tumour transplantation. Control mice received PBS injections (Figure 4A). The body weight of 4T1‐bearing mice began to decline around Day 16 and showed a statistically significant reduction compared to controls by Day 24. This weight loss was effectively attenuated by OH‐CATH30 treatment, resulting in a clear difference between the OH‐CATH30–treated group and the 4T1‐only group (Figure 4B). Additionally, while food intake was reduced in 4T1‐bearing mice, OH‐CATH30 treatment partially restored consumption (Figure 4C).

OH‐CATH30 also prevented the decline in grip strength observed in 4T1 tumour‐bearing mice, suggesting its potential to protect against muscle weakness associated with cachexia (Figure 4D). Importantly, no significant difference in tumour growth was observed between OH‐CATH30‐treated and control groups (Figure S10), indicating that the effects of OH‐CATH30 were specifically directed at muscle wasting, not tumour progression. Muscle mass loss in the GA muscle was prominent in 4T1 tumour‐bearing mice, but OH‐CATH30 treatment significantly preserved muscle mass (Figure 4E). Histological analysis through H&E staining revealed that OH‐CATH30 effectively countered the reduction in myofiber CSA in the GA muscles (Figure 4F). These results highlight the ability of OH‐CATH30 to alleviate muscle wasting in a tumour‐induced cachexia model.

### 3.6 OH‐CATH30 Alleviates Cisplatin‐Induced Muscle Atrophy

**FIGURE 5:**
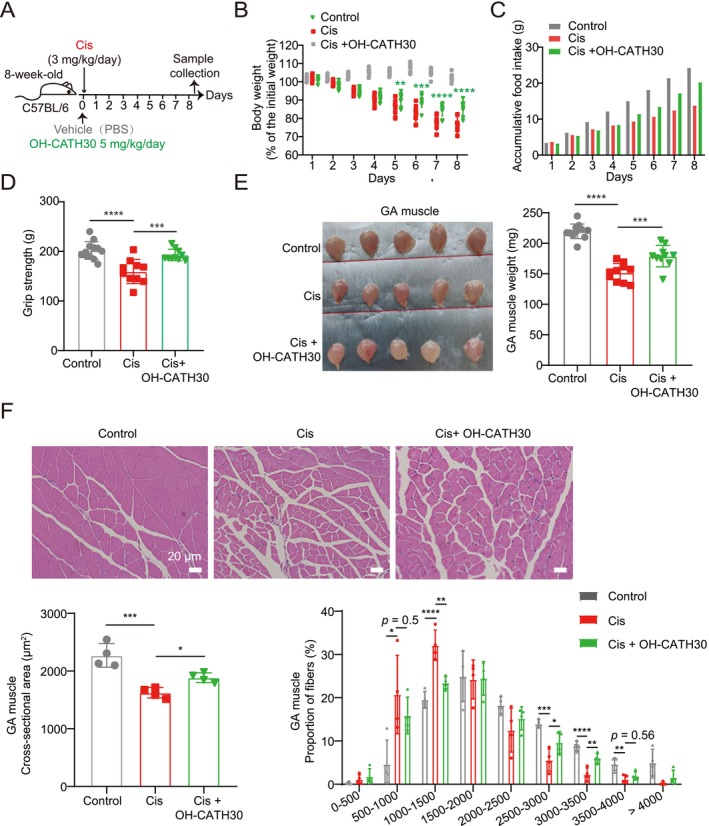
Effects of OH‐CATH30 on cisplatin‐challenged mice. (A) Schematic diagram of the experimental design: 8‐week‐old male C57BL/6 mice received cisplatin (3 mg/kg/day, ip) for 8 days, with simultaneous daily administration of OH‐CATH30 (5 mg/kg, ip) or PBS. Body weight and food intake were measured daily. *n* = 10 per group. (B) Body weight of each group mice. (C) Average accumulative food intake of each group mice. (D) Grip strength of each group mice. (E) Representative images and weights of GA muscles for each group of mice. (F) Representative photomicrographs of H&E‐stained gastrocnemius muscle sections and cross‐sectional area (CSA) from each group mice. Scale bar: 20 μm. *n* = 4. Values are mean ± SD. Significance determined using one‐way ANOVA (B, D, E and F). *p < 0.05, **p < 0.01, ***p < 0.001 and ****p < 0.0001 versus cisplatin‐treated group.

To investigate whether OH‐CATH30 can mitigate cisplatin‐induced muscle atrophy, we administered OH‐CATH30 to mice 30 min before cisplatin treatment over a 10‐day period (Figure 5A). Cisplatin treatment led to significant weight loss, which was markedly reduced in the OH‐CATH30‐treated group (Figure 5B). Furthermore, cisplatin administration resulted in decreased food intake, which was partially restored by OH‐CATH30 treatment (Figure 5C), suggesting that OH‐CATH30 may prevent loss of appetite. To assess muscle function and mass, we measured grip strength, muscle weight and fibre CSA. Cisplatin‐treated mice exhibited significant muscle weakness, reflected by reduced grip strength, which was significantly improved in the OH‐CATH30‐treated group (Figure 5D). Additionally, cisplatin caused a reduction in GA muscle weight and fibre CSA, but OH‐CATH30 treatment preserved both muscle weight and myofiber size (Figure 5E,F).

Together, all of these results provide compelling evidence that OH‐CATH30 effectively protects against muscle wasting across different cachexia models, highlighting its potential as a therapeutic strategy for managing cachexia‐associated muscle atrophy.

### 3.7 OH‐CATH30 Suppresses Inflammation and Protein Degradation While Enhancing Cellular Metabolism Across Cachexia Models

To elucidate the molecular mechanisms by which OH‐CATH30 mitigates muscle atrophy across cachexia models, we performed transcriptomic analysis of GA muscle from LPS‐induced, 4T1 tumour‐induced and cisplatin‐induced cachexia models treated with OH‐CATH30. Differential gene expression analysis revealed that OH‐CATH30 modulated key pathways involved in inflammation, protein degradation, muscle structure, and cellular metabolism, potentially contributing to reduced muscle wasting and enhanced cellular stability (Figures S11 and 6 and Table S7).

**FIGURE 6:**
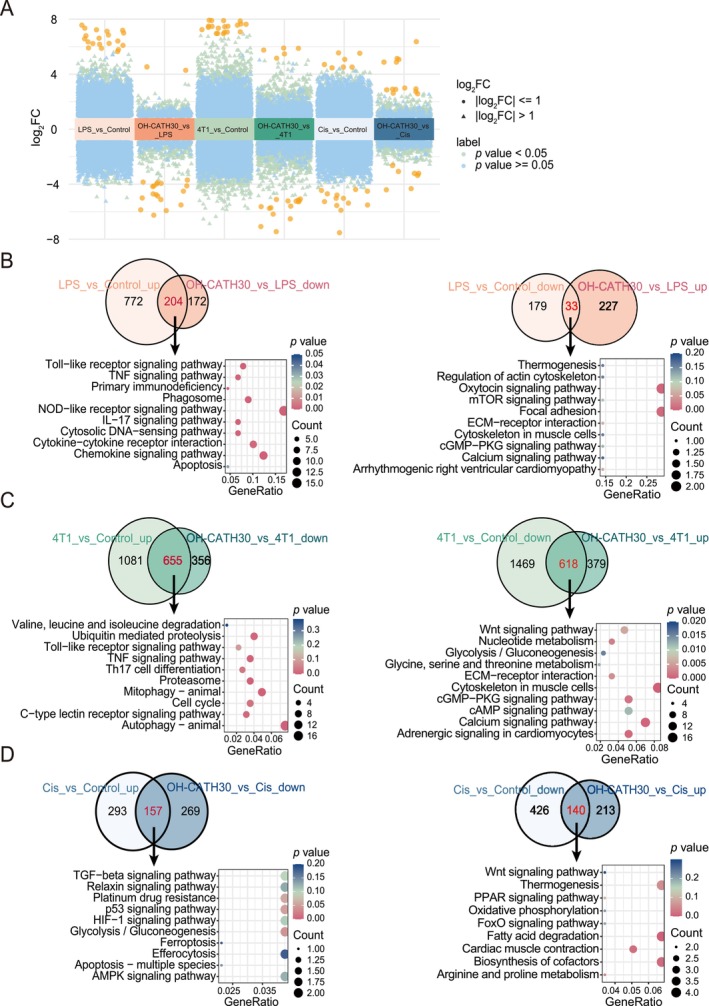
Transcriptomic analysis of OH‐CATH30's effects in cachexia models. (A) Volcano plots of differentially expressed genes comparing each condition: Control vs. model (LPS‐induced sepsis, 4T1 tumour‐induced cancer cachexia, Cisplatin‐induced chemotherapy‐associated cachexia) and OH‐CATH30 vs. each model group. Each dot represents a gene, with significantly upregulated and downregulated genes highlighted (*p* < 0.05, |log2FC| > 1). Orange dots represent the top 20 genes with the highest |log_2_ fold change| in each comparison group. (B) Venn diagrams and KEGG enrichment of genes in the LPS‐induced sepsis model. Left: Genes upregulated in the model (LPS_vs_Control_up) and downregulated with OH‐CATH30 treatment (OH‐CATH30_vs_LPS_down). Right: Genes downregulated in the model (LPS_vs_Control_down) and upregulated with OH‐CATH30 treatment (OH‐CATH30_vs_LPS_up). KEGG pathways significantly enriched in these intersecting genes are visualized with bubble plots. (C) Venn diagrams and KEGG enrichment for the 4T1 tumour‐induced cancer cachexia model. Left: Genes upregulated in the model (4T1_vs_Control_up) and downregulated with OH‐CATH30 treatment (OH‐CATH30_vs_4T1_down). Right: Genes downregulated in the model (4T1_vs_Control_down) and upregulated with OH‐CATH30 treatment (OH‐CATH30_vs_4T1_up). KEGG enrichment is displayed with bubble plots. (D) Venn diagrams and KEGG enrichment for the cisplatin‐induced chemotherapy‐associated cachexia model. Left: Genes upregulated in the model (Cis_vs_Control_up) and downregulated with OH‐CATH30 treatment (OH‐CATH30_vs_Cis_down). Right: Genes downregulated in the model (Cis_vs_Control_down) and upregulated with OH‐CATH30 treatment (OH‐CATH30_vs_Cis_up). Bubble plots illustrate KEGG pathways enriched in these intersecting genes.

In the LPS‐induced cachexia model, OH‐CATH30 treatment downregulated 204 genes that were upregulated by LPS, predominantly associated with inflammatory pathways such as TLR signalling, TNF signalling and IL‐17 signalling, indicating a suppression of LPS‐induced inflammatory responses. Additionally, OH‐CATH30 upregulated 33 genes downregulated by LPS, which were associated with cytoskeleton organization, mTOR signalling and calcium signalling pathways, suggesting enhanced cytoskeletal stability and restoration of muscle cell function (Figure 6A,B). In the 4T1 tumour‐induced cachexia model, OH‐CATH30 downregulated 655 genes upregulated by 4T1 tumour presence, primarily related to protein degradation pathways, including ubiquitin‐mediated proteolysis and autophagy. Additionally, OH‐CATH30 upregulated 618 genes downregulated by 4T1, linked to pathways associated with cellular metabolism, such as Wnt signalling and glycolysis/gluconeogenesis, which are indicative of increased metabolic activity and improved muscle cell structural integrity (Figure 6A,C). In the cisplatin‐induced cachexia model, OH‐CATH30 treatment downregulated 157 genes upregulated by cisplatin exposure, which were associated with stress‐response pathways including TGF‐β signalling, p53 signalling and ferroptosis, pointing to a reduction in chemotherapy‐induced cellular stress and apoptosis. Concurrently, OH‐CATH30 upregulated 140 genes suppressed by cisplatin, associated with pathways such as Wnt signalling and oxidative phosphorylation, suggesting a potential restoration of muscle cell metabolic function (Figure 6A,D).

### 3.8 Validation of OH‐CATH30's Effects on Inflammation and Protein Degradation in 4T1‐Induced Cachexia

**FIGURE 7:**
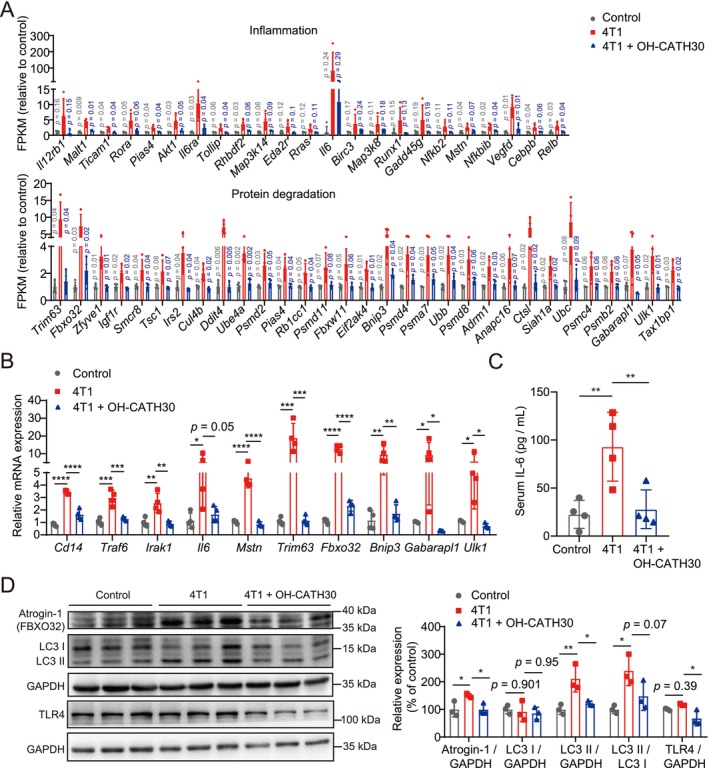
Validation of OH‐CATH30's effects in 4T1‐induced cachexia model. (A) Bar chart of differentially expressed genes involved in inflammation and protein degradation in GA muscle of control, 4T1 and 4T1 + OH‐CATH30 groups. Genes associated with inflammation (e.g., *Il6ra*, *Nfkbib*, *Relb*) and protein degradation (e.g., *Fbxo32*, *Trim63*, *Bnip3*) are shown. *n* = 4–5. (B) Quantitative PCR analysis of selected genes associated with TLR4‐mediated inflammation (*Cd14*, *Traf6*, *Irak1*, *Il6*) and protein degradation (*Mstn*, *Trim63*, *Fbxo32*, *Bnip3*, *Gabarapl1*, *Ulk1*). *n* = 4. (C) Serum IL‐6 levels across the three groups, measured by ELISA. *n* = 4. (D) Western blot analysis of TLR4, Atrogin‐1 (FBXO32) and autophagy markers LC3 I and LC3 II in GA muscle tissue, with GAPDH as a loading control. Densitometric quantification of TLR4/GAPDH, Atrogin‐1/GAPDH, LC3 I/GAPDH and LC3 II/LC3 I ratios is provided. *n* = 3. Values are mean ± SD. Significance determined using one‐way ANOVA (B‐D). *p < 0.05, **p < 0.01, ***p < 0.001 and ****p < 0.0001 versus 4T1 group.

To validate transcriptomic findings on OH‐CATH30's effects, we examined its impact on inflammation and protein degradation in the 4T1 tumour‐induced cachexia model. Differential gene expression analysis showed significant upregulation of inflammatory genes (e.g., *Il6*, *Mstn*, *Il1b*, *Tnf* and *Ifng*) and protein degradation genes (e.g., *Fbxo32*, *Trim63* and *Bnip3*) in the GA muscle of 4T1 tumour‐bearing mice. Treatment with OH‐CATH30 partially reversed these gene expression changes (Figure 7A and Table S8). qPCR further confirmed the downregulation of key inflammatory markers (*Il6* and *Mstn*) and protein degradation markers (*Trim63*, *Fbxo32*, *Bnip3*, *Gabarapl1* and *Ulk1*) in OH‐CATH30‐treated muscle compared to the 4T1 group (Figure 7B).

Serum analysis revealed elevated IL‐6 levels in 4T1 tumour‐bearing mice, indicative of systemic inflammation, which were significantly reduced by OH‐CATH30 treatment (Figure 7C), suggesting an attenuation of systemic inflammatory response. Western blot analysis demonstrated that OH‐CATH30 downregulated Atrogin‐1 (FBXO32) expression and modulated autophagy markers LC3 I and LC3 II (Figure 7D), indicating a reduction in protein degradation within skeletal muscle.

Notably, OH‐CATH30 treatment significantly suppressed the transcription of *Cd14*, *Traf6* and *Irak1* (Figure 7B) and reduced TLR4 protein levels in skeletal muscle of cachectic mice (Figure 7D), further supporting the involvement of TLR4‐dependent signalling in mediating its anti‐atrophic effects.

Collectively, these results demonstrate that OH‐CATH30 exerts protective effects across cachexia models by suppressing TLR4‐mediated inflammatory pathways, reducing protein degradation and enhancing cellular metabolism, thereby mitigating muscle atrophy and preserving muscle cell function.

### 3.9 Pharmacologic TLR4 Blockade Recapitulates the Protective Effects of OH‐CATH30 and Exhibits Non‐Additive Efficacy In Vitro and In Vivo

To further assess whether the anti‐atrophic effects of OH‐CATH30 are mediated through TLR4 signalling, we conducted parallel pharmacological inhibition experiments using TAK‐242, a selective TLR4 inhibitor, in both C2C12 myotube and an LPS‐induced cachexia mouse model.

In vitro, differentiated C2C12 myotubes were treated with TNF‐α in the presence of vehicle, OH‐CATH30, TAK‐242 or the combination of both agents (Figure S12A). TNF‐α stimulation induced pronounced myotube atrophy, evidenced by loss of MyHC expression and fibre fragmentation. Both OH‐CATH30 and TAK‐242 individually preserved MyHC protein levels and maintained myotube integrity (Figure S12B,C). Notably, co‐treatment with OH‐CATH30 and TAK‐242 failed to provide additional protection beyond monotherapy (Figure S12B,C), suggesting a convergent mechanism of action.

**FIGURE 8:**
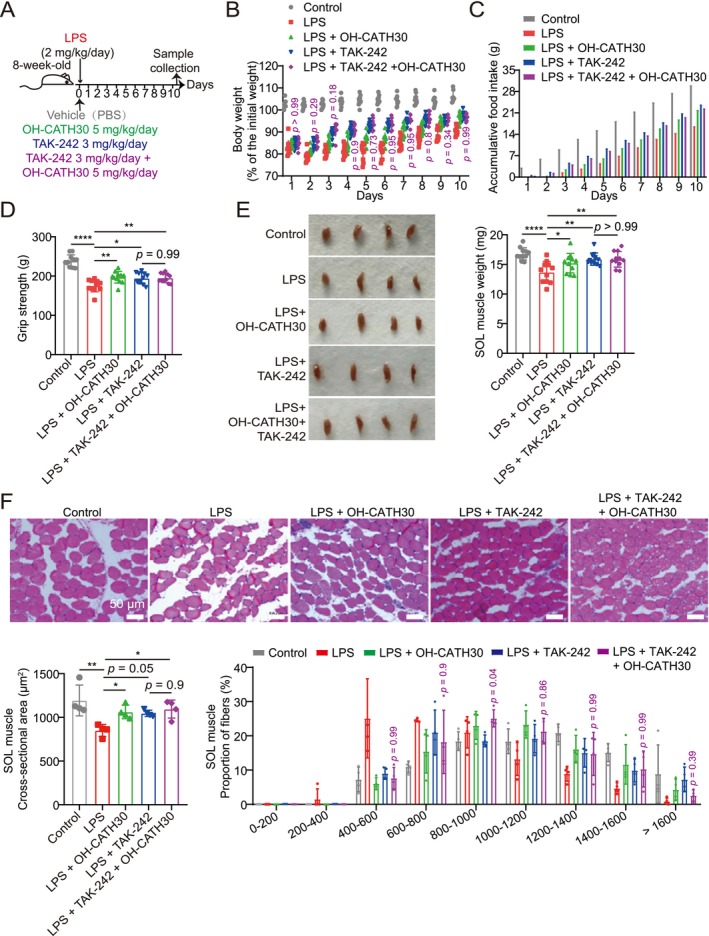
Pharmacologic TLR4 inhibition by TAK‐242 phenocopies the anti‐cachectic effects of OH‐CATH30 in LPS‐challenged mice. (A) Experimental design: Eight‐week‐old male C57BL/6 mice were intraperitoneally injected with LPS (2 mg/kg/day) for 10 consecutive days and concurrently treated with vehicle (PBS), OH‐CATH30 (5 mg/kg/day), TAK‐242 (3 mg/kg/day), or a combination of both agents. (B) Body weight of each group mice. *n* = 11. (C) Average accumulative food intake of each group mice. (D) Grip strength of each group mice. *n* = 11. (E) Representative images and weights of SOL muscles for each group of mice. *n* = 11. (F) Representative photomicrographs of H&E‐stained soleus muscle sections and CSA from each group mice. *n* = 4. Scale bar: 50 μm. Values are mean ± SD. Significance determined using one‐way ANOVA (B, D, E and F). *p < 0.05, **p < 0.01 and ****p < 0.0001 versus LPS‐treated or TAK‐242‐treated group.

In vivo, male C57BL/6 mice were administered LPS for 10 consecutive days and co‐treated with vehicle, OH‐CATH30, TAK‐242, or both (Figure 8A). LPS challenge induced body weight loss followed by partial recovery. Both OH‐CATH30 and TAK‐242 improved weight regain and food intake relative to the LPS group, while the combination treatment did not confer additive benefits (Figure 8B,C). Similarly, each treatment independently restored forelimb grip strength, SOL muscle mass and myofiber CSA; however, co‐administration did not enhance these effects (Figure 8D–F).

Collectively, these findings demonstrate that pharmacological inhibition of TLR4 recapitulates the morphological, functional, and structural benefits of OH‐CATH30 in both cellular and animal models. The consistent absence of synergistic effects between TAK‐242 and OH‐CATH30 suggests that the protective actions of OH‐CATH30 are primarily mediated via TLR4‐dependent signalling pathways.

## 4 Discussion

Cachexia, a multifactorial syndrome characterized by severe muscle wasting and weight loss, is prevalent in chronic diseases, including cancer, sepsis and following chemotherapy [1]. This syndrome poses significant clinical challenges as it contributes to increased morbidity and mortality, especially in cancer patients [1]. Despite the prevalence of cachexia, effective treatments remain limited, with most interventions focusing on symptom management rather than directly addressing the molecular mechanisms underlying muscle wasting. This study aims to address this gap by exploring the TLR4 signalling pathway as a key regulator of inflammation‐driven muscle wasting across multiple cachexia models, along with the therapeutic potential of a novel TLR4 inhibitory peptide, OH‐CATH30 [21], derived from snake venom.

Our transcriptomic analysis shows that the TLR4 signalling pathway serves as a core mediator in sepsis, cancer cachexia and chemotherapy‐induced cachexia models. As an innate immune receptor, TLR4 recognizes pathogen‐associated molecular patterns (PAMPs) such as bacterial endotoxins, which trigger inflammatory responses [23, 24]. While TLR4 activation aids host defence under normal conditions, its dysregulation can lead to tissue damage. In cachexia, abnormal TLR4 activation promotes excessive release of inflammatory cytokines, including TNF‐α and IL‐6 [25], which are known to drive muscle protein breakdown and atrophy [26, 27]. Our findings indicate that TLR4 is upregulated across cachexia models, establishing a chronic inflammatory state that accelerates muscle protein degradation. Previous studies have reported that specific TLR4 knockout in mouse skeletal muscle can reduce inflammation and protein degradation levels, thereby alleviating muscle atrophy caused by lung cancer‐induced cachexia and improving mouse survival [13]. Combining our research with these findings, inhibiting TLR4 to reduce inflammation and protein degradation presents a promising strategy for counteracting muscle wasting associated with cachexia.

To investigate targeted TLR4 inhibition, we evaluated OH‐CATH30, which has been shown to bind specifically to TLR4 and suppress sepsis‐induced inflammation [21]. Our in vitro and in vivo studies assessed OH‐CATH30's effects on muscle atrophy, inflammatory cytokine production, and markers of muscle protein degradation. Results demonstrated that OH‐CATH30 effectively mitigated cachexia‐induced muscle wasting, reducing levels of key pro‐inflammatory cytokines (TNF‐α, IL‐6) and downregulating muscle‐specific proteolytic enzymes in both the ubiquitin‐proteasome and autophagy‐lysosome pathways. Unlike studies limited to a single model, our research tested OH‐CATH30 across sepsis, cancer, and chemotherapy‐induced cachexia, revealing its broad‐spectrum efficacy in reducing muscle atrophy and highlighting its potential as a versatile therapeutic for cachexia.

Although our data suggest that OH‐CATH30 exerts anti‐cachectic effects partly via modulation of TLR4 signalling, we acknowledge that OH‐CATH30 is a pleiotropic peptide that may modulate multiple pattern recognition receptors. Prior studies have shown its ability to inhibit TLR2 responses in skin [28] and suggested that TLR4 contributes only partially to its systemic effects [21]. Thus, while our results implicate TLR4 as a mechanistic target in the context of muscle atrophy, further studies are warranted to dissect the relative contributions of other TLRs and innate immune pathways in mediating the full spectrum of OH‐CATH30 activity.

Peptides hold significant promise as drug candidates due to their high selectivity, low toxicity and favourable biocompatibility [14, 15]. Compared to small molecules, peptides generally exhibit greater target specificity, enabling selective binding to specific receptors or enzymes and minimizing effects on non‐target tissues, often resulting in fewer side effects [14, 15, 29]. We believe OH‐CATH30 exemplifies these advantages and represents a viable option for treating cachexia‐related muscle atrophy. However, as a peptide drug, the translational application of OH‐CATH30 faces several hurdles common to peptide‐based therapies, including limited bioavailability, susceptibility to enzymatic degradation, and challenges in large‐scale manufacturing. For clinical application, these issues must be addressed to ensure consistent therapeutic levels in patients. Novel drug delivery systems, such as encapsulation in exosomes or nanoparticles [30], may enhance OH‐CATH30's stability and prolong its release, thus reducing dosing frequency. Furthermore, chemical modifications to increase its resistance to enzymatic degradation could enhance its clinical viability. Addressing these challenges will be critical for advancing OH‐CATH30 from preclinical studies to clinical trials and eventual therapeutic use.

Although this study primarily focuses on the anti‐inflammatory and anti–protein degradation effects of OH‐CATH30, we believe its protective mechanisms against muscle wasting may extend beyond these pathways. Cachexia‐related chronic inflammation is often accompanied by oxidative stress, mitochondrial dysfunction, and metabolic imbalance, all of which contribute to skeletal muscle degradation [1, 2]. Notably, our transcriptomic data suggest that under cachectic conditions, OH‐CATH30 may exert beneficial effects by improving glucose and lipid metabolism as well as enhancing mitochondrial oxidative phosphorylation in skeletal muscle.

This hypothesis is further supported by findings from the cisplatin‐induced cachexia model. Despite the lack of pronounced activation of TLR4 signalling pathways in this model, OH‐CATH30 still markedly ameliorated body weight loss and skeletal muscle atrophy. This suggests that the protective effect of OH‐CATH30 in this context may not rely solely on TLR4 inhibition but may involve alternative signalling pathways. Future studies should investigate whether OH‐CATH30 modulates additional mechanisms, such as TGF‐β signalling, metabolic reprogramming or mitochondrial homeostasis, to confer its protective effects against cisplatin‐induced muscle wasting and better elucidate its therapeutic potential.

In the 4T1‐induced cachexia model, OH‐CATH30 treatment was initiated before significant body weight loss was observed. This early intervention was designed to assess the preventive potential of OH‐CATH30. However, we recognize that therapeutic treatment during the later stages of cachexia would better reflect clinical intervention scenarios. Future studies should include delayed administration paradigms to assess the therapeutic efficacy of OH‐CATH30 once cachexia is fully established.

It is worth noting that OH‐CATH30 belongs to the cathelicidin family of antimicrobial peptides [21, 31], with LL‐37 being the primary member of this family in humans [32]. Similar to OH‐CATH30, LL‐37 possesses significant anti‐inflammatory properties [33] and has shown therapeutic potential in inflammatory and autoimmune diseases, including arthritis [34], psoriasis [35], systemic lupus erythematosus [36] and sepsis [37]. While the benefits of LL‐37 in these conditions are well documented, its role in cachexia‐related muscle atrophy remains largely unexplored. Given the connection between chronic inflammation and muscle wasting in cachexia, LL‐37's anti‐inflammatory effects may make it a promising therapeutic candidate. Investigating LL‐37's role in cachexia‐induced muscle wasting could deepen our understanding of the inflammation‐muscle metabolism relationship and uncover its potential as a treatment strategy. Alongside OH‐CATH30, exploring LL‐37 as a therapeutic option represents a valuable research direction that may expand treatment options for cachexia‐induced muscle wasting, ultimately improving patient outcomes and quality of life.

This study, while promising, has certain limitations that warrant further investigation. Our findings are primarily based on animal models, which may not fully replicate the complexity of cachexia in human patients. Differences in immune response and metabolism between species could affect the translatability of our results to clinical settings. Future studies should consider using patient‐derived cells or organoid models to validate OH‐CATH30's efficacy in human‐like environments. Additionally, conducting longer‐term studies in animal models could provide insights into the sustainability of OH‐CATH30's effects and its safety profile over extended periods, which are essential for developing it as a viable therapeutic option. Lastly, while our findings suggest a TLR4‐mediated mechanism for OH‐CATH30, the absence of TLR4 knockout models limits the strength of causal inference. Although TAK‐242 treatment phenocopied OH‐CATH30's effects both in vitro and in vivo, genetic validation would provide more definitive mechanistic evidence. Future studies should incorporate TLR4‐deficient or tissue‐specific knockout models to confirm the direct involvement of this pathway.

In conclusion, this study identifies TLR4 signalling as a common driver of cachexia and demonstrates the potential of OH‐CATH30 as a therapeutic peptide to alleviate muscle wasting across diverse models, offering a novel strategy for targeting inflammation‐driven muscle atrophy in chronic diseases (Figure S13).

## Conflicts of Interest

The authors declare no conflicts of interest.

## Supporting information


**Figure S1:** Construction of LPS‐induced sepsis, 4T1 tumor‐induced cancer cachexia, and cisplatin‐induced chemotherapy‐associated cachexia models. (A) Schematic diagram of the experimental design of 8‐week‐old mice treated with LPS, 4T1 tumor and cisplatin. (B) Body weight and muscle weights (gastrocnemius [GA], tibialis anterior [TA] and soleus [SOL]) in LPS‐induced sepsis, 4T1 tumor‐induced cancer cachexia and cisplatin‐induced chemotherapy‐associated cachexia models. Values are mean ± SD. Significance determined using unpaired *t* test. *p < 0.05, **p < 0.01, ***p < 0.001 and ****p < 0.0001 versus control group.
**Figure S2:** Principal component analysis (PCA) of transcriptomic data from the GA muscle in control and model groups for three cachexia models: LPS‐induced sepsis, 4T1 tumor‐induced cancer cachexia and cisplatin‐induced chemotherapy‐associated cachexia.
**Figure S3:** Related to Figure 1. (A) Protein–protein interaction (PPI) network of genes significantly enriched in the pathways identified in Figure 1B for each of the three cachexia models. Node color and size represent the degree of connectivity, while edge thickness and transparency indicate the combined score, reflecting the interaction strength between genes. (B) Bar chart showing the top 14–15 upregulated genes with the highest degree in the PPI network for each model, highlighting the clustering of inflammation‐related genes.
**Figure S4:** FPKM analysis of TLR4 signaling–related gene expression in the LPS‐induced cachexia model. Values are mean ± SD. Significance determined using unpaired t test.
**Figure S5:** FPKM analysis of TLR4 signaling–related gene expression in the 4T1 tumor‐induced cachexia model. Values are mean ± SD. Significance determined using unpaired t test.
**Figure S6:** FPKM analysis of TLR4 signaling–related gene expression in the cisplatin‐induced cachexia model. Values are mean ± SD. Significance determined using unpaired t test.
**Figure S7:** Structure and amino acid composition of OH‐CATH30. The structure of OH‐CATH30 was predicted by Alpha‐fold3 and visualized by ChimerX1.8.
**Figure S8:** Transcriptomics analysis of the TNF‐α‐treated and control C2C12 myotubes. (A) PCA of transcriptomic data from TNF‐α‐treated and control C2C12 myotubes, showing distinct clustering between the two groups. (B) Volcano plots visualizing differential expression analysis results from the transcriptomic data of TNF‐α‐treated versus control C2C12 myotubes, highlighting significantly upregulated and downregulated genes.
**Figure S9:** Effects of OH‐CATH30 on 4T1 cell supernatant‐ and cisplatin‐treated C2C12 myotubes. (A) Schematic diagram of the experimental design showing C2C12 myotubes treated with 4T1 cell supernatant and OH‐CATH30 (0–5 μg/mL) for 48 h. (B) Schematic diagram of the experimental design showing C2C12 myotubes treated with cisplatin (150 μM) and OH‐CATH30 (0–5 μg/mL) for 48 h. (C and D) Western blot analysis of MyHC protein expression in 4T1 cell supernatant‐treated (C) and cisplatin‐treated (D) C2C12 myotubes in the presence of OH‐CATH30. (E and F) H&E staining of myotube structures in 4T1 cell supernatant‐treated (E) and cisplatin‐treated (F) C2C12 myotubes. Scale bar: 100 μm.
**Figure S10:** Tumor weight comparison between the 4T1 and 4T1 + OH‐CATH30‐treated groups in mice. Values are mean ± SD. Significance determined using unpaired t test.
**Figure S11:** PCA of transcriptomic data from the three models, comparing the control group, model group (LPS‐induced sepsis, 4T1 tumor‐induced cancer cachexia, and cisplatin‐induced chemotherapy‐associated cachexia) and OH‐CATH30 treatment group.
**Figure S12:** Pharmacologic TLR4 inhibition by TAK‐242 phenocopies OH‐CATH30 in TNF‐α–induced C2C12 myotube atrophy. (A) Schematic diagram of the experimental design. Differentiated C2C12 myotubes were treated with TNF‐α (10 ng/mL) in the presence of vehicle, OH‐CATH30 (5 μg/mL), TAK‐242 (1 μM) or their combination for 48 h. (B) Western blot analysis of MyHC protein expression in each treatment group. (C) Immunofluorescence staining and quantification of myotube morphology. Scale bar: 100 μm. n = 3. Values are mean ± SD. Significance determined using one‐way ANOVA (C). *p < 0.05, **p < 0.01 and ****p < 0.0001 versus vehicle‐treated or TAK‐242‐trated group.
**Figure S13:** Proposed mechanism of OH‐CATH30 in protecting against distinct cachexia‐induced skeletal muscle atrophy via TLR4 signaling modulation.
**Table S1:** Primer sequences for qPCR.

**Table S2:** Supporting information.

**Table S3:** Supporting information.

**Table S4:** Supporting information.

**Table S5:** Supporting information.

**Table S6:** Supporting information.

**Table S7:** Supporting information.

**Table S8:** Supporting information.

## Data Availability

All data supporting the findings of this study are available in the main text or in the Supporting Information. All transcriptomics data generated for this study are available at the National Genomics Data Center of the China National Center for Bioinformation with the following accession numbers: CRA028198.
