# Supplementary material for: Peptide OH‐CATH30 Mitigates Cachexia‐Induced Muscle Atrophy via Modulation of TLR4‐Associated Inflammation
Source: J Cachexia Sarcopenia Muscle. 2026 Jan 29;17(1):e70195. doi: 10.1002/jcsm.70195 (PMC12856054; doi:10.1002/jcsm.70195)
Supplement: Supplementary file 1 — Figure S1: Construction of LPS‐induced sepsis, 4T1 tumor‐induced cancer cachexia, and cisplatin‐induced chemotherapy‐associated cachexia models. (A) Schematic diagram of the experimental design of 8‐week‐old mice treated with LPS, 4T1 tumor and cisplatin. (B) Body weight and muscle weights (gastrocnemius [GA], tibialis anterior [TA] and soleus [SOL]) in LPS‐induced sepsis, 4T1 tumor‐induced cancer cachexia and cisplatin‐induced chemotherapy‐associated cachexia models. Values are mean ± SD. Significance determined using unpaired t test. *p < 0.05, **p < 0.01, ***p < 0.001 and ****p < 0.0001 versus control group. Figure S2: Principal component analysis (PCA) of transcriptomic data from the GA muscle in control and model groups for three cachexia models: LPS‐induced sepsis, 4T1 tumor‐induced cancer cachexia and cisplatin‐induced chemotherapy‐associated cachexia. Figure S3: Related to Figure 1. (A) Protein–protein interaction (PPI) network of genes significantly enriched in the pathways identified in Figure 1B for each of the three cachexia models. Node color and size represent the degree of connectivity, while edge thickness and transparency indicate the combined score, reflecting the interaction strength between genes. (B) Bar chart showing the top 14–15 upregulated genes with the highest degree in the PPI network for each model, highlighting the clustering of inflammation‐related genes. Figure S4: FPKM analysis of TLR4 signaling–related gene expression in the LPS‐induced cachexia model. Values are mean ± SD. Significance determined using unpaired t test. Figure S5: FPKM analysis of TLR4 signaling–related gene expression in the 4T1 tumor‐induced cachexia model. Values are mean ± SD. Significance determined using unpaired t test. Figure S6: FPKM analysis of TLR4 signaling–related gene expression in the cisplatin‐induced cachexia model. Values are mean ± SD. Significance determined using unpaired t test. Figure S7: Structure and amino acid composition of OH‐CATH30. The [file JCSM-17-e70195-s001.docx]

**Supplementary materials**

**Supplementary Methods**

**Reagents**

OH-CATH30 (KFFKKLKNSVKKRAKKFFKKPRVIGVSIPF) were synthesized by GL Biochem (Shanghai, China). TNF-α (CF09) was purchased from novoprotein (Suzhou, China). LPS (L2630) and cisplatin (232120) were obtained from Sigma-Aldrich Chemical Co. (St. Louis, MO, USA). TAK-242 (HY-11109) was purchased form MedChemExpress (Princeton, NJ, USA). Dulbecco’s modified Eagle’s minimal essential medium (DMEM) (high glucose, C3113-0500), RPMI 1640, Foetal bovine serum (FBS, C04001-500) and trypsin/EDTA (C3530-0100) were purchased from Biological Industries (BI) (Kibbutz Beit Haemek, Israel). TRIzol reagent (9109), Prime Script RT-PCR and SYBR® Premix Ex Taq™ (Tli RNaseH Plus) kits (RR047A) were attained from TaKaRa Bio (Beijing, China).

**Cell culture and OH-CATH30 treatment**

The 4T1 mouse breast cancer cell line was obtained from the Shanghai Cell Bank, Chinese Academy of Sciences and cultured in growth medium at 37°C with 5% CO2. When 95% confluent, cells were washed, incubated in serum-free DMEM for 24 hours, and the conditioned medium (4T1CM) was collected, centrifuged, and stored at -80°C. To test OH-CATH30's protective effects, differentiated myotubes were co-incubated with 4T1CM and OH-CATH30 (0, 2.5, 5 μg/mL) for 48 hours. The medium was replaced daily during the treatment.

**Grip strength measurement**

Forelimb grip strength was measured using a grip strength meter (KW-ZL-1, Karwin, Nanjing, China). Mice were lifted by their tails to grasp the meter's grid, and gently pulled back until they released their grip, recording the peak tension as grip strength. Each mouse completed five trials, and the average of the maximum values (in grams) was calculated.

**RNA-sequencing and analysis**

RNA-sequencing were performed at Novogene Co., Ltd. (Beijing, China). For RNA-seq, 1 μg of total RNA was processed using the TrueLib mRNA Library Prep Kit for Illumina (ExCell Bio). mRNA was isolated, fragmented, and reverse transcribed into cDNA, which was then end-repaired, barcoded with adapters, and used to construct a cDNA library. The libraries were purified with AmpureXP beads, quantified with Qubit (Invitrogen), and sequenced on an Illumina HiSeq platform. RNA-seq data were analyzed in RStudio (genek, <http://gs63.genek.cn:8787/>) using the DEGseq2 package to identify differentially expressed transcripts (*p* < 0.05 and |log2FC| ≥ 1). Specifically, the PCA was performed on a filtered dataset: genes with zero counts across all samples and genes with zero variance were removed prior to analysis. KEGG pathway enrichment was performed with the clusterProfiler package and visualized using enrich plot.

**Antibodies of Western blotting**

The primary antibodies included mouse anti-MyHC (MF20) (1:75, DSHB, Iowa City, IA, United States), mouse anti-GAPDH (1:20000, 10366-1-AP, Proteintech, Wuhan, China), mouse anti-MAFbx (1:1000, sc-166806, Santa Cruz Biotechnology, Dallas, TX, USA), mouse anti-LC3 (1:1000, 14600-1-AP, Proteintech, Wuhan, China), mouse anti-TLR4 (1:1000, PTM-5192, PTM Bio, Hangzhou, China). Secondary antibodies were HRP-conjugated goat anti-mouse (1:10000, GB23301, Servicebio, Wuhan, China) and goat anti-rabbit (1:10000, GB23303, Servicebio, Wuhan, China).

**Immunofluorescent staining**

To assess myotube diameter after OH-CATH30 treatment, C2C12 myotubes were washed with PBS (three times), fixed in 4% paraformaldehyde for 20 minutes, and permeabilized with 0.02% Triton-X100 for 15 minutes at room temperature. Blocking was performed with 0.5% BSA in PBS. Cells were then incubated overnight at 4°C with MYH2 antibody (1:50, sc-53095, Santa Cruz Biotechnology, Dallas, TX, USA). After washing with PBS, they were incubated with FITC-labeled goat anti-rabbit secondary antibody (1:200, ab150077, Abcam, Cambridge, MA, USA) for 1 hour at room temperature. Following three final washes, myotube images were captured using an Image Xpress Micro 4 High-Content Imaging System, and their width was measured using ImageJ software.

**Haematoxylin-eosin staining**

Myotubes were washed with PBS for 3 times, fixed with 4% paraformaldehyde for 1 h, and then washed with cold PBS for 3 times. The fixed muscle tissue samples were embedded in paraffin and sectioned into 5-μm thickness. Cell and Tissue samples were stained with hematoxylin and eosin staining by standard procedures and visualized by an optical microscopy (zeiss, German). Randomly selected at least 10 images from individual mouse each group to assess the cross-sectional area of all myofibers, which quantified on H&E-stained tissue sections by using Image J.

**Measurement of inflammatory cytokines in serum**

The serum samples of mice were collected and stored at −80°C. For the analysis of IL-6 levels in the serum samples, the mouse IL-6 ELISA Kit (KE10091) from Proteintech group (Wuhan, China) were used, and the analysis was conducted according to the manufacturer's instructions.

**Pharmacological inhibition of TLR4 by TAK-242**

To assess the contribution of TLR4 signaling to the anti-atrophic effects of OH-CATH30, we employed TAK-242, a selective TLR4 inhibitor, in both *in vitro* and *in vivo* models. *In vitro*, fully differentiated C2C12 myotubes were treated with TNF-α (10 ng/mL) for 48 hours to induce atrophy, in the presence or absence of OH-CATH30 (5 μg/mL), TAK-242 (1 μM), or their combination. Myotube morphology and MyHC protein levels were subsequently analyzed.

*In vivo*, LPS-induced cachexia was modeled in 8-week-old male C57BL/6 mice (n = 11 per group). Mice were randomly assigned to one of five groups: control (PBS), LPS, LPS + OH-CATH30, LPS + TAK-242, and LPS + OH-CATH30 + TAK-242. LPS was administered intraperitoneally at a dose of 2 mg/kg/day for 10 consecutive days to induce muscle atrophy. Concurrently, mice received daily intraperitoneal injections of OH-CATH30 (5 mg/kg/day), TAK-242 (3 mg/kg/day), or vehicle (PBS). Body weight and food intake were recorded daily. At the end of the treatment period, skeletal muscles were harvested for histological, molecular, and biochemical analyses as described above.

**Supplementary Figures**


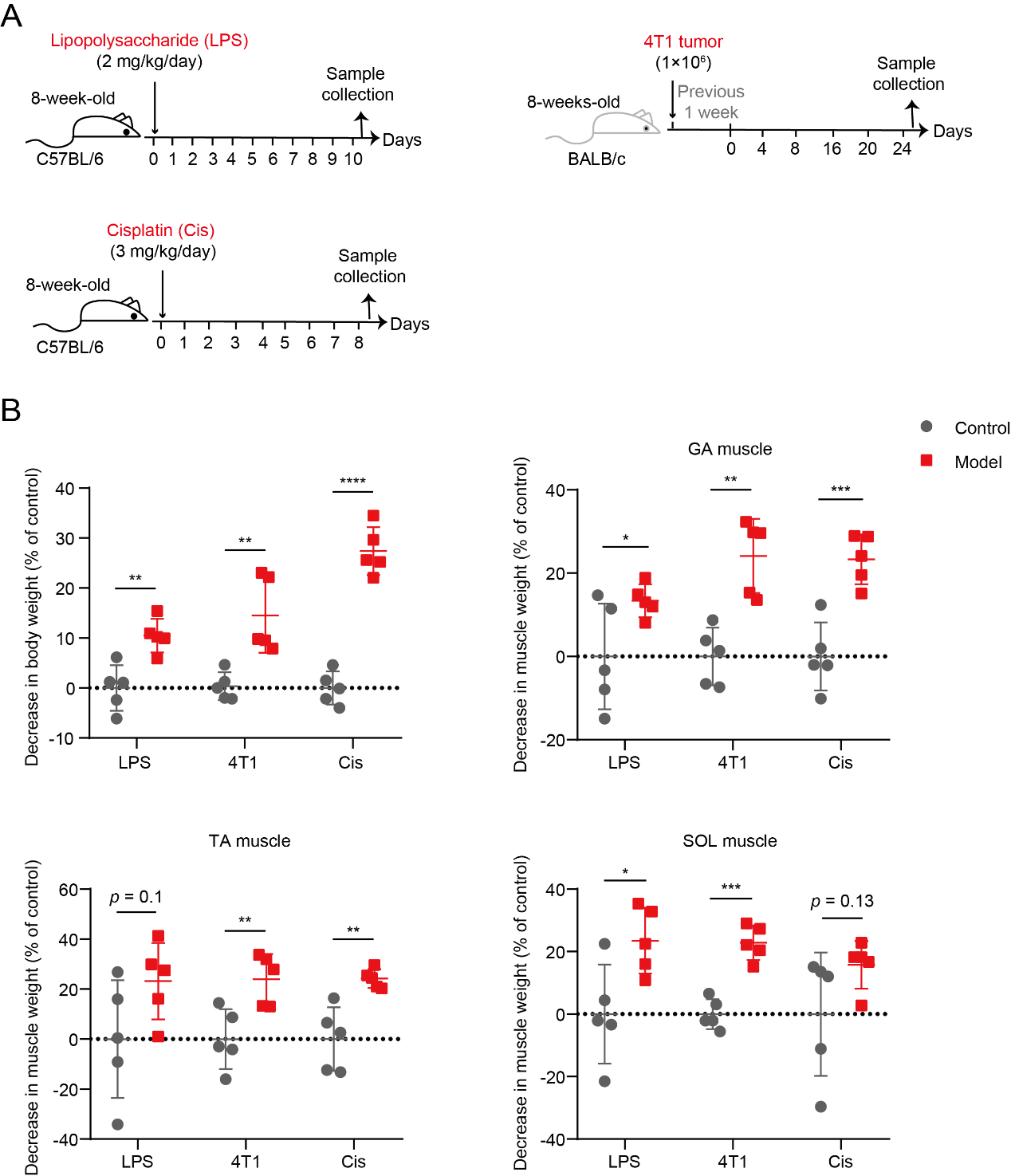


**Supplementary Figure 1. Construction of LPS-induced sepsis, 4T1 tumor-induced cancer cachexia, and cisplatin-induced chemotherapy-associated cachexia models. (A)** Schematic diagram of the experimental design of 8-week-old mice treated with LPS, 4T1 tumor and cisplatin. **(B)** Body weight and muscle weights (gastrocnemius (GA), tibialis anterior (TA), and soleus (SOL)) in LPS-induced sepsis, 4T1 tumor-induced cancer cachexia, and cisplatin-induced chemotherapy-associated cachexia models. Values are mean ± SD. Significance determined using unpaired *t* test. **p* < 0.05, ***p* < 0.01, ****p* < 0.001 and *****p* < 0.0001 versus control group.


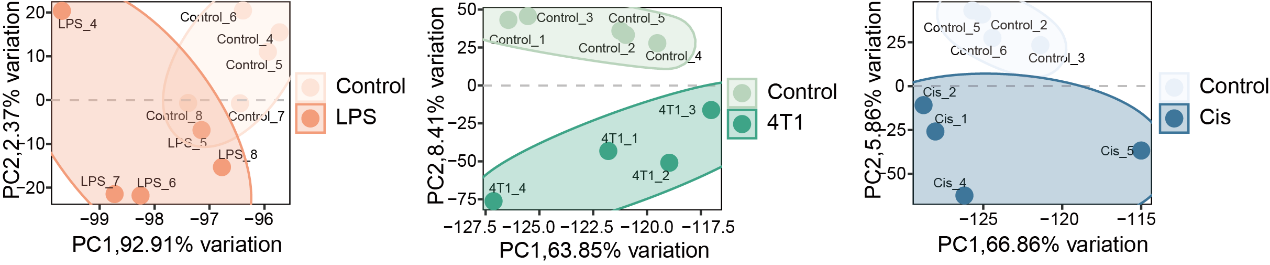


**Supplementary figure 2**. **Principal component analysis (PCA) of transcriptomic data from the GA muscle in control and model groups for three cachexia models: LPS-induced sepsis, 4T1 tumor-induced cancer cachexia, and cisplatin-induced chemotherapy-associated cachexia.**


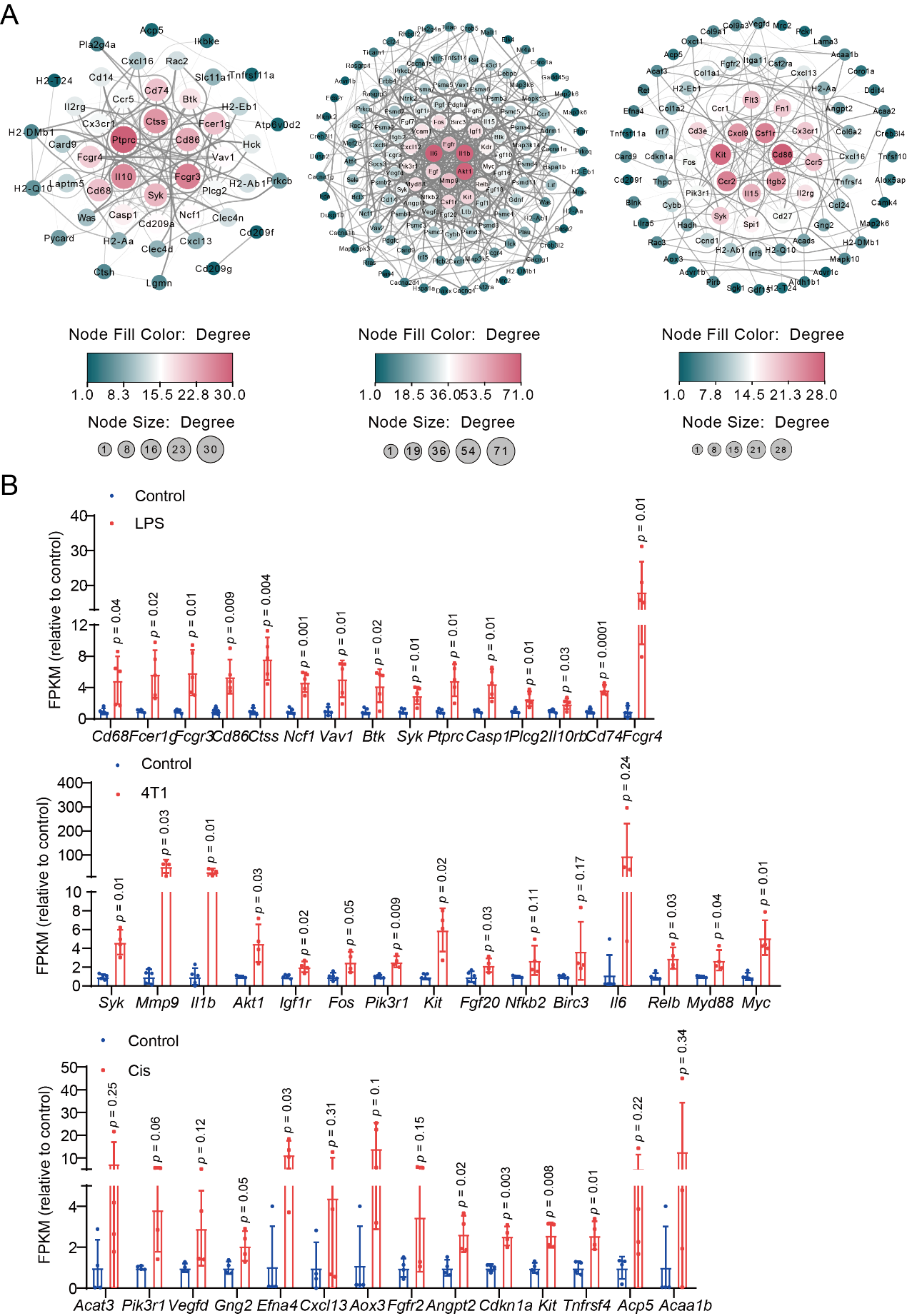


**Supplementary figure 3**. **Related to Figure 1. (A)** Protein–protein interaction (PPI) network of genes significantly enriched in the pathways identified in Figure 1B for each of the three cachexia models. Node color and size represent the degree of connectivity, while edge thickness and transparency indicate the combined score, reflecting the interaction strength between genes. **(B)** Bar chart showing the top 14-15 upregulated genes with the highest degree in the PPI network for each model, highlighting the clustering of inflammation-related genes.


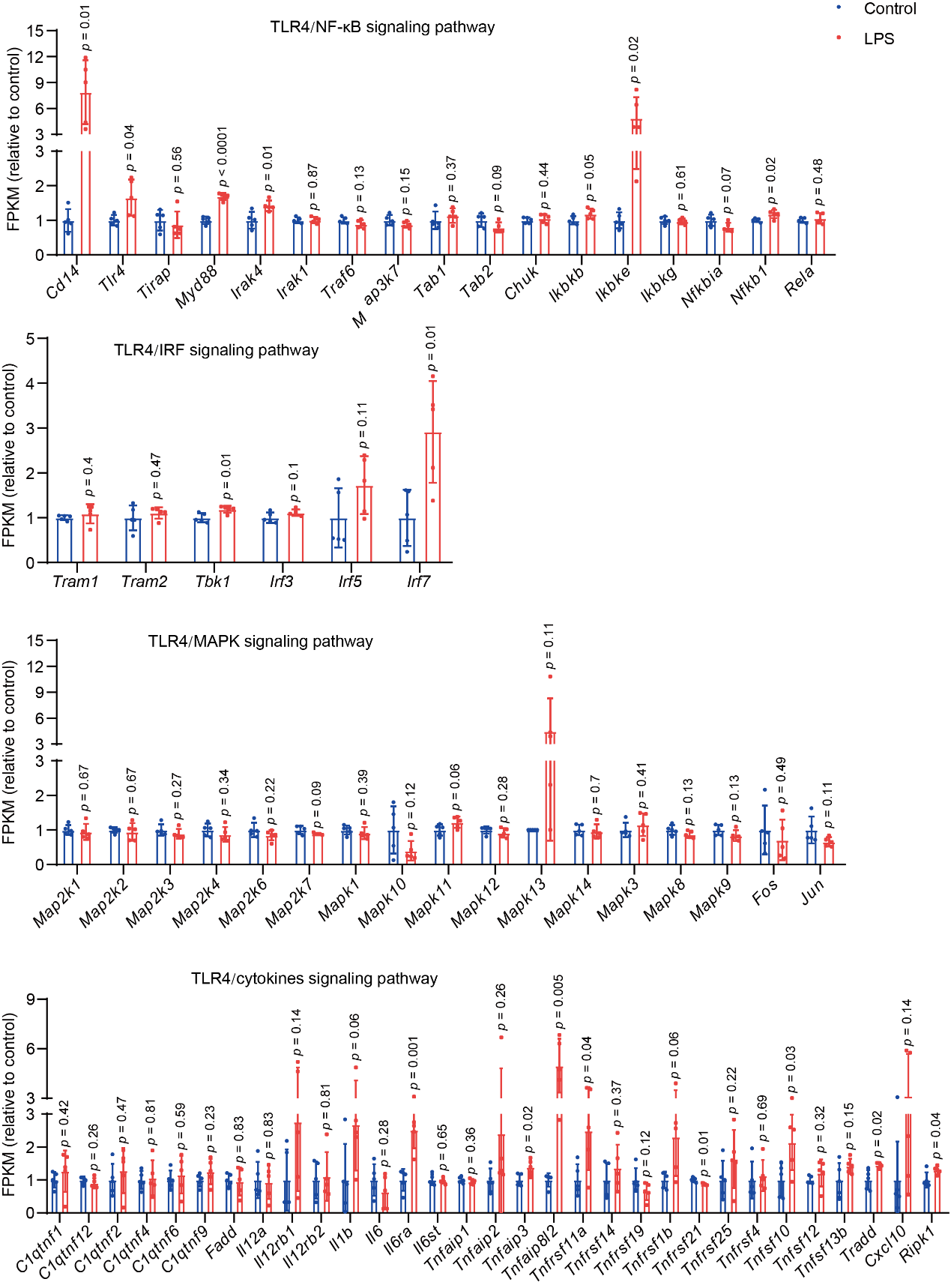


**Supplementary figure 4**. **FPKM analysis of TLR4 signaling–related gene expression in the LPS-induced cachexia model.** Values are mean ± SD. Significance determined using unpaired *t* test.


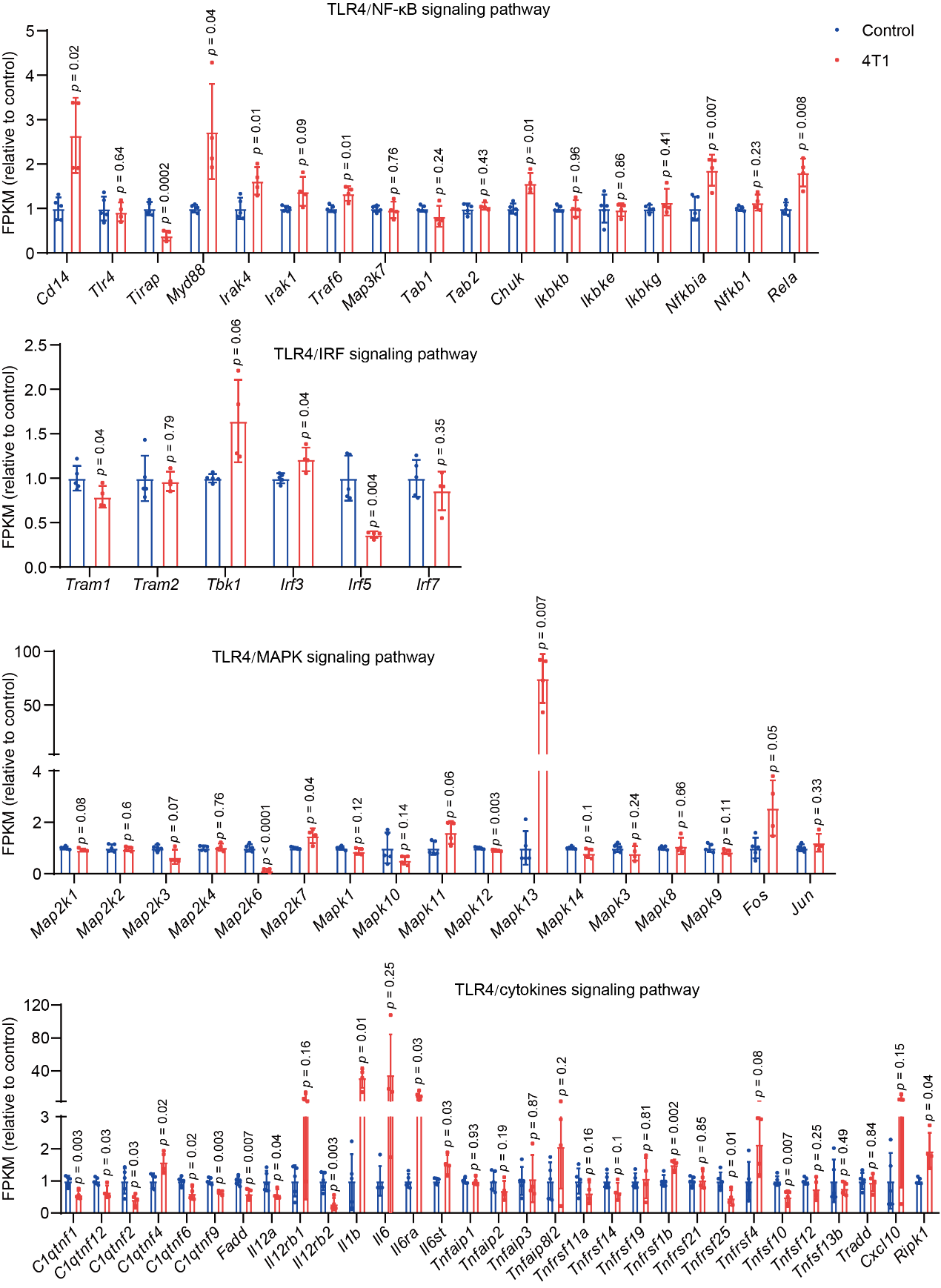


**Supplementary figure 5. FPKM analysis of TLR4 signaling–related gene expression in the 4T1 tumor-induced cachexia model.** Values are mean ± SD. Significance determined using unpaired *t* test.


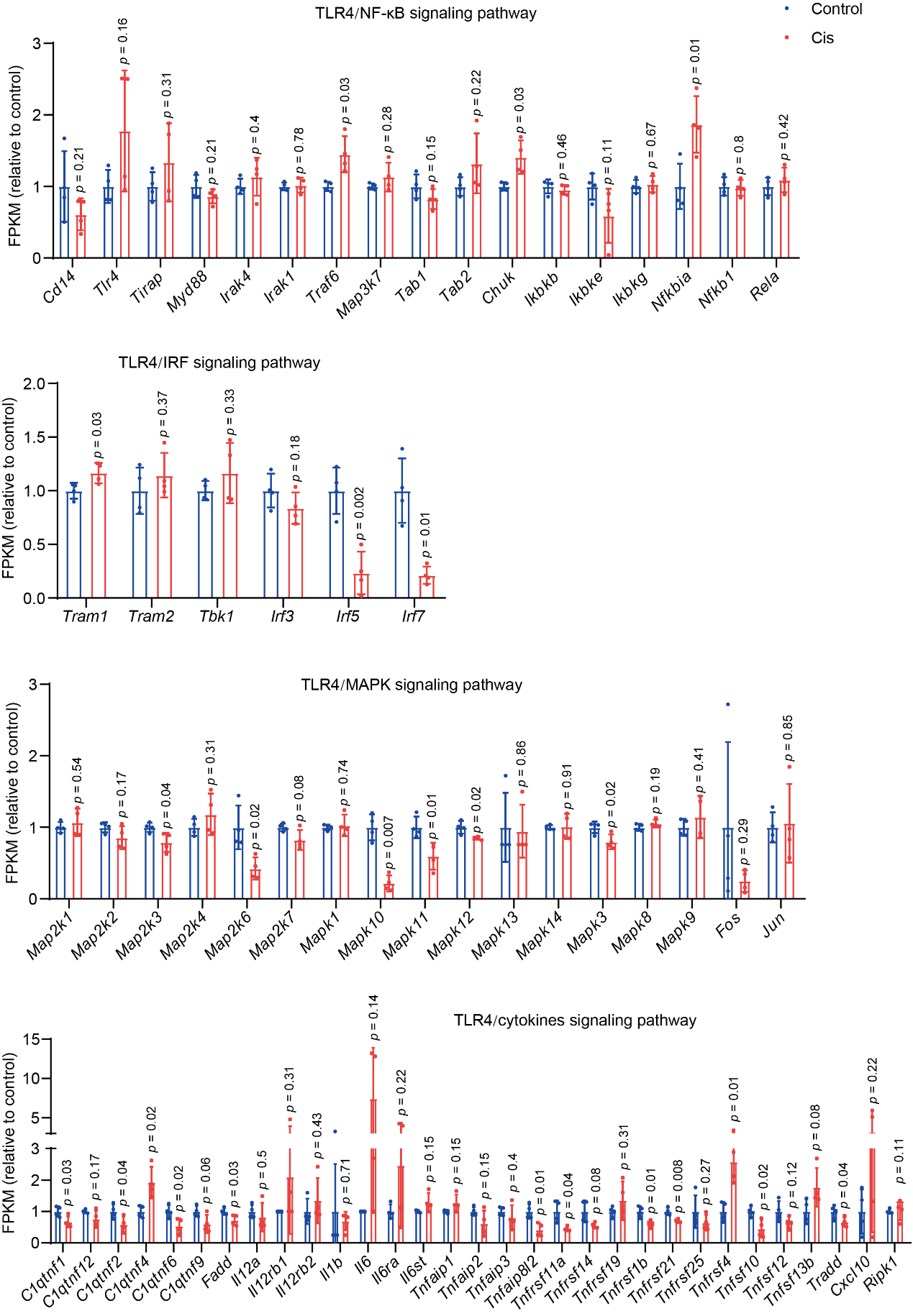


**Supplementary figure 6. FPKM analysis of TLR4 signaling–related gene expression in the cisplatin-induced cachexia model.** Values are mean ± SD. Significance determined using unpaired *t* test.

**
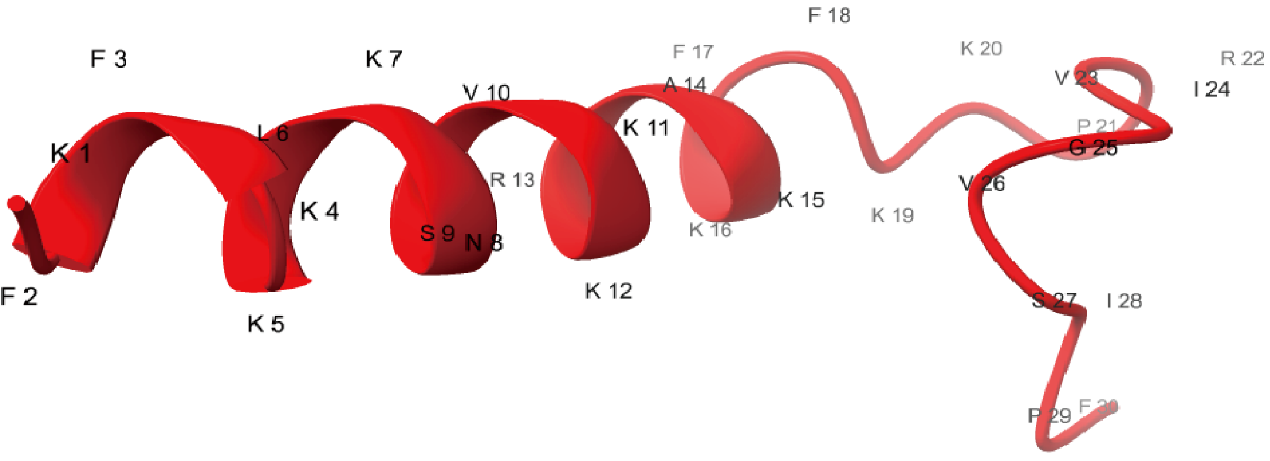
**

**Supplementary figure 7**. **Structure and amino acid composition of OH-CATH30.** The structure of OH-CATH30 was predicted by Alpha-fold3 and visiulazed by ChimerX1.8.


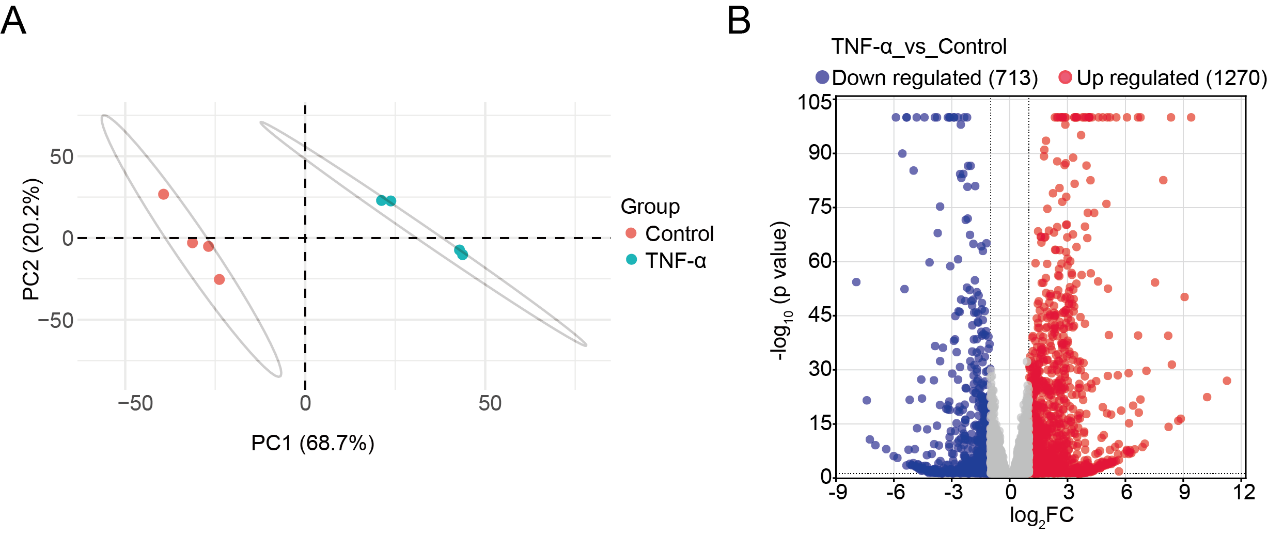


**Supplementary figure 8**. **Transcriptomics analysis of the TNF-α-treated and control C2C12 myotubes.** **(A)** PCA of transcriptomic data from TNF-α-treated and control C2C12 myotubes, showing distinct clustering between the two groups. **(B)** Volcano plots visualizing differential expression analysis results from the transcriptomic data of TNF-α-treated versus control C2C12 myotubes, highlighting significantly upregulated and downregulated genes.


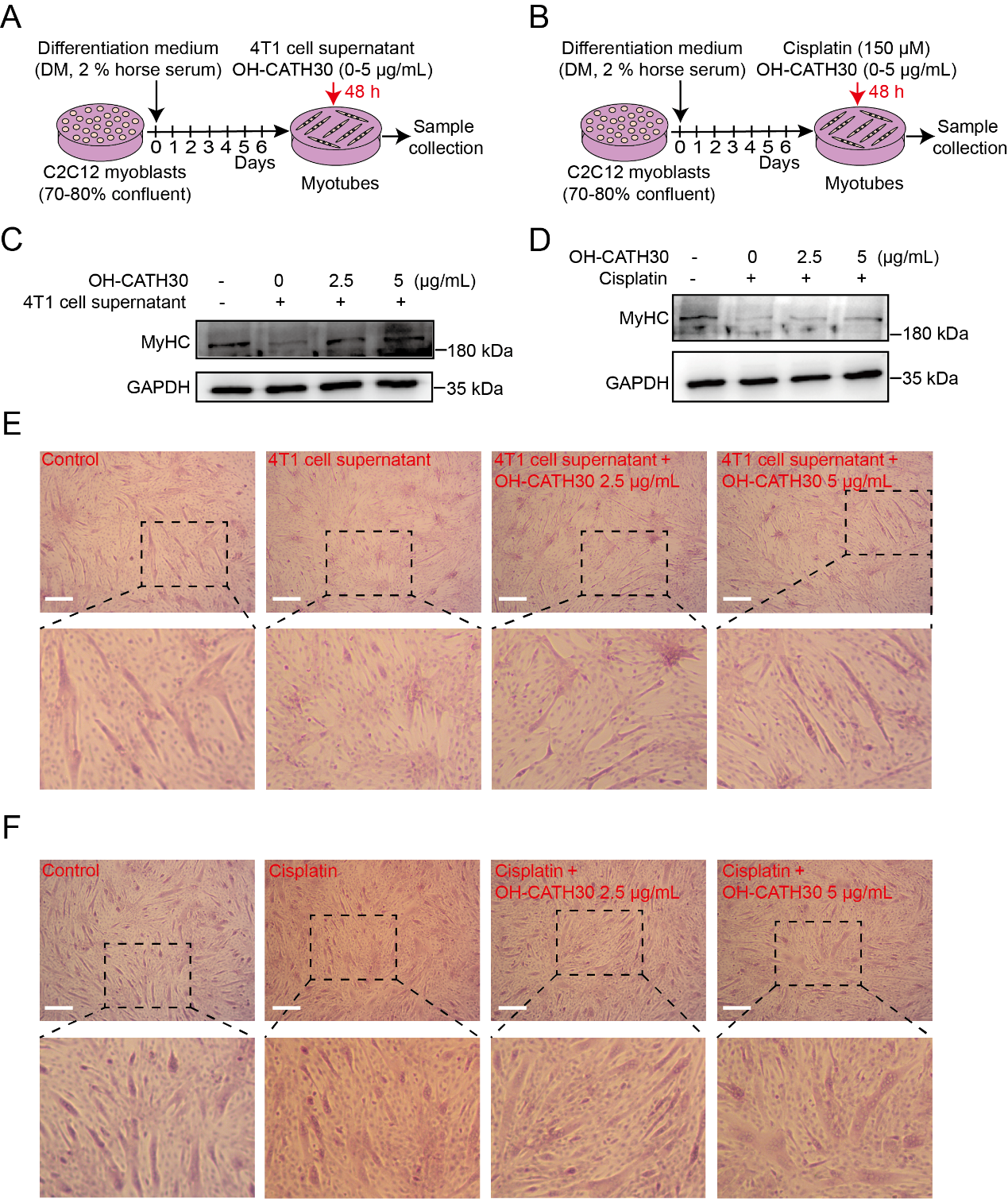


**Supplementary figure 9**. **Effects of OH-CATH30 on 4T1 cell supernatant- and cisplatin-treated C2C12 myotubes.** **(A)** Schematic diagram of the experimental design showing C2C12 myotubes treated with 4T1 cell supernatant and OH-CATH30 (0–5 μg/mL) for 48 hours. **(B)** Schematic diagram of the experimental design showing C2C12 myotubes treated with cisplatin (150 μM) and OH-CATH30 (0–5 μg/mL) for 48 hours. **(C and D)** Western blot analysis of MyHC protein expression in 4T1 cell supernatant-treated (C) and cisplatin-treated (D) C2C12 myotubes in the presence of OH-CATH30. **(E and F)** H&E staining of myotube structures in 4T1 cell supernatant-treated (E) and cisplatin-treated (F) C2C12 myotubes. Scale bar: 100 μm.


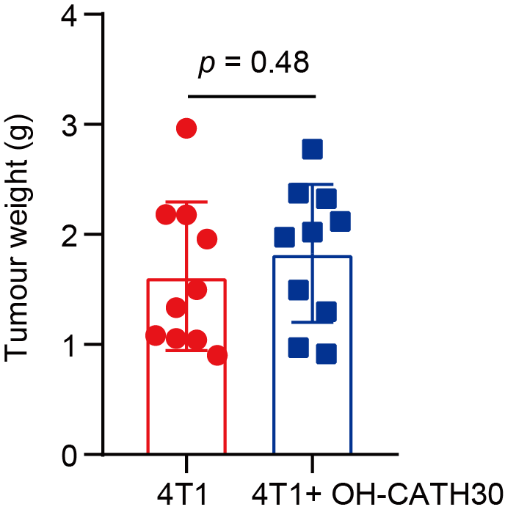


**Supplementary figure 10**. **Tumor weight comparison between the 4T1 and 4T1 + OH-CATH30-treated groups in mice.** Values are mean ± SD. Significance determined using unpaired *t* test.


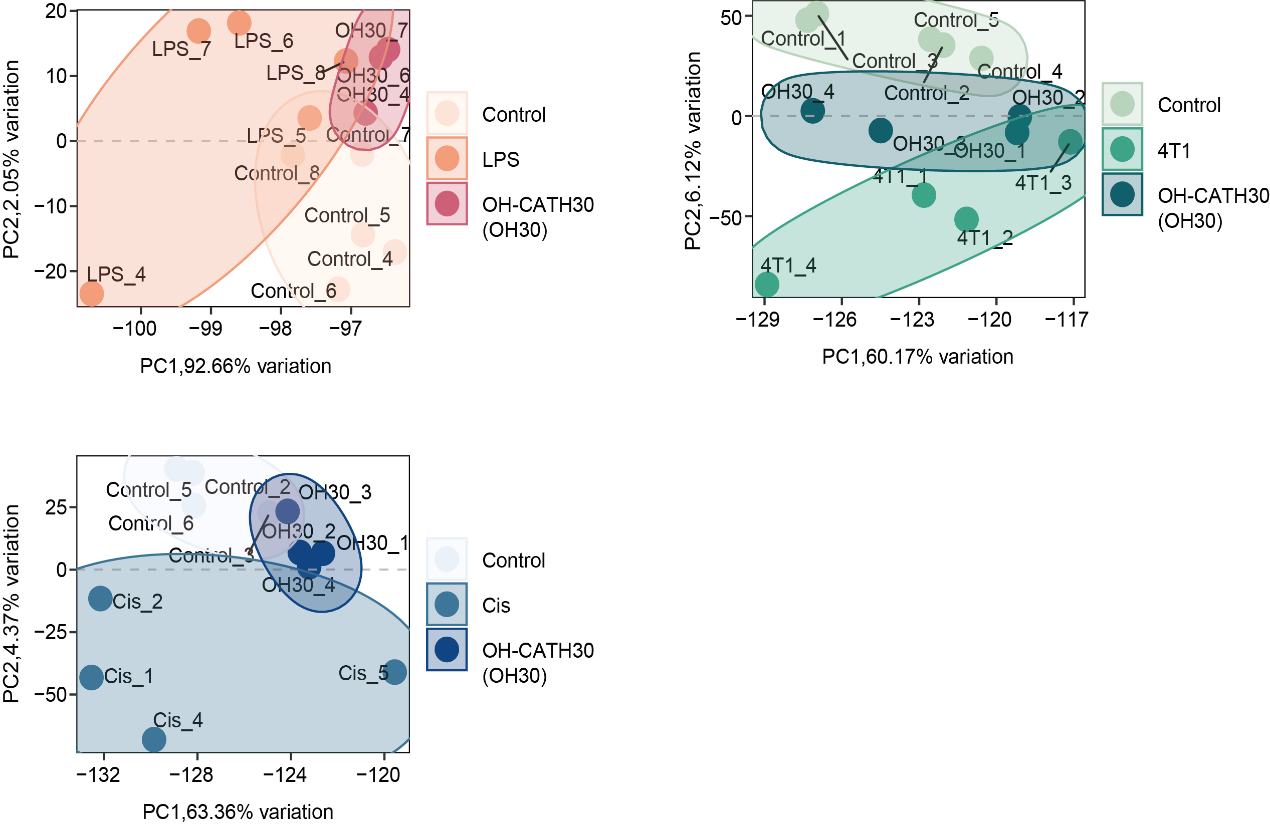


**Supplementary figure 11**. **PCA of transcriptomic data from the three models, comparing the control group, model group (LPS-induced sepsis, 4T1 tumor-induced cancer cachexia, and cisplatin-induced chemotherapy-associated cachexia), and OH-CATH30 treatment group.**


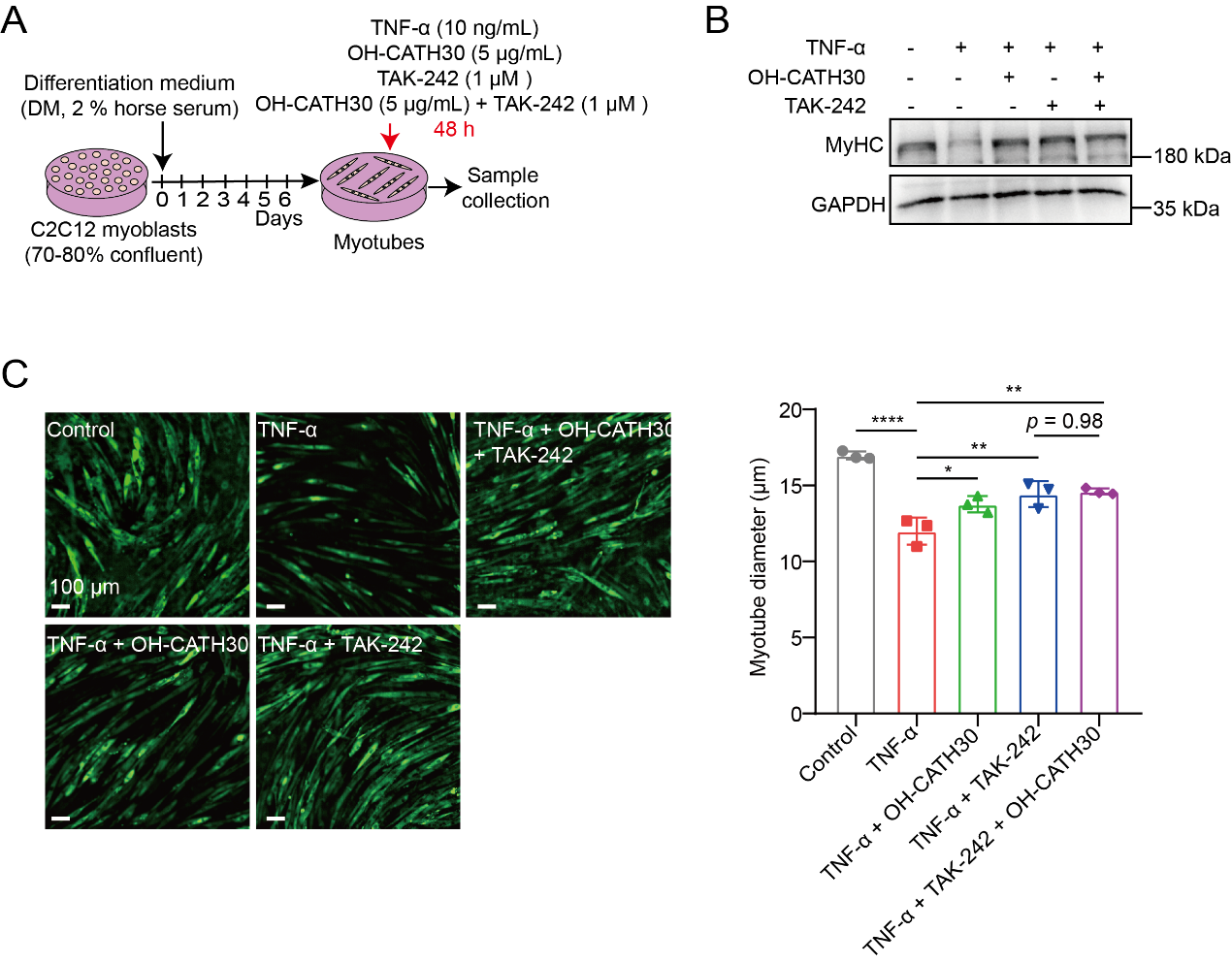


**Supplementary figure 12. Pharmacologic TLR4 inhibition by TAK-242 phenocopies OH-CATH30 in TNF-α–induced C2C12 myotube atrophy**. **(A)** Schematic diagram of the experimental design. Differentiated C2C12 myotubes were treated with TNF-α (10 ng/mL) in the presence of vehicle, OH-CATH30 (5 μg/mL), TAK-242 (1 μM), or their combination for 48 hours. **(B)** Western blot analysis of MyHC protein expression in each treatment group. **(C)** Immunofluorescence staining and quantification of myotube morphology. Scale bar: 100 μm. n = 3. Values are mean ± SD. Significance determined using one-way ANOVA (C). **p* < 0.05, ***p* < 0.01 and *****p* < 0.0001 versus vehicle-treated or TAK-242-trated group.


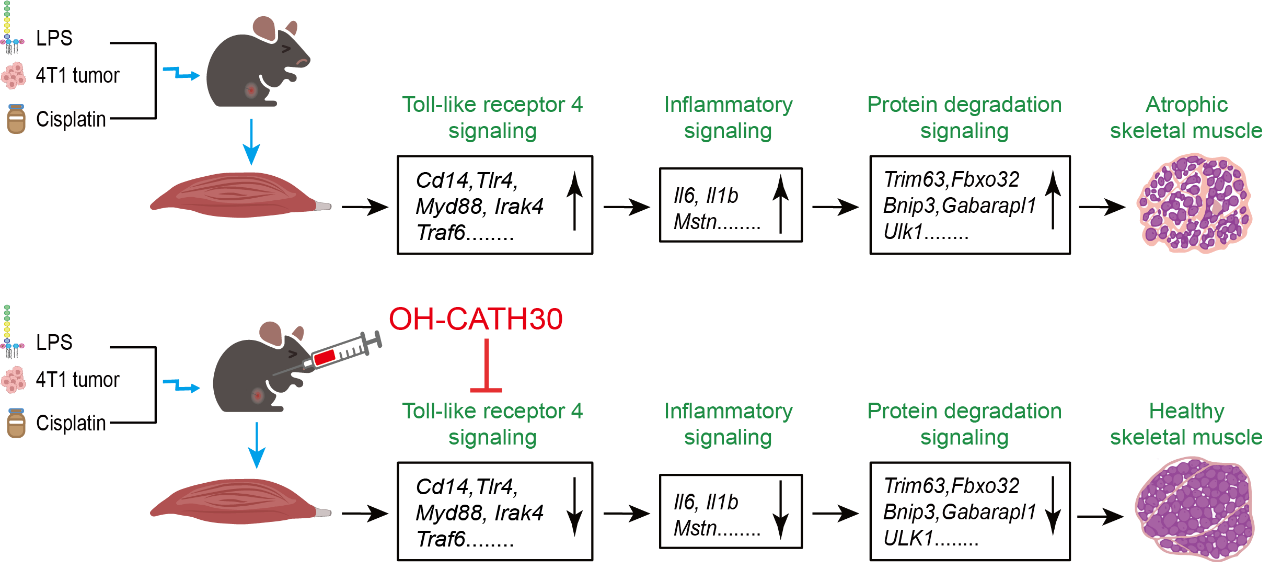


**Supplementary figure 13. Proposed mechanism of OH-CATH30 in protecting against distinct cachexia-induced skeletal muscle atrophy via TLR4 signaling modulation.**

**Supplementary Table S1. Primer sequences for qPCR**

| Gene | Primer sequence(5’→3’) |
| --- | --- |
| *Mstn* | F: GCACTGGTATTTGGCAGAGT |
| *Il6*  *Fbxo32*  *Trim63*  *CD14*  *Irak1*  *Irak4*  *Traf6*  *Tlr4*  *Bnip3*  *Gabarapl1*  *Ulk1*  *Gapdh*  *C1qtnf4*  *Il6ra*  *Mapk13*  *Rela*  *Tbk1*  *Ikbke*  *Irf7*  *Tnfaip8l2*  *Chuk*  *Fos*  *Tnfrsf4*  *Tram1*  *Il1b*  *Ikbke*  *Nfkbia* | R: TTCAGCCCATCTTCTCCTGG  F: CTCCCAACAGACCTGTCTATAC  R: CCATTGCACAACTCTTTTCTCA  F: GCAAACACTGCCACATTCTCTC  R: CTTGAGGGGAAAGTGAGACG  F: TGACCACAGAGGGTAAAG  R: TGTCTCACTCATCTCCTTCTTC  F: CTCTGTCCTTAAAGCGGCTTAC  R: GTTGCGGAGGTTCAAGATGTT  F: GGCTCAACTAGCTTGCTGCT  R: TAGTGCCTCCCTGGGTACAG  F: AGCTGCGTCACCTACCTGTT  R: GTTTGGTGATGTTGCTGTGG  F: CAAACCAGAACTGCTTGCCT  R: GCATCAGTACTTCGTGGCTG  F: CAGCAGAGGAAGAACAAGAA  R: TGCAAACAGACTGGGTTTAG  F: GGGTTTTCCCCAAAGGAATA  R: GAATCCTCATCCTGCAAAGC  F: CATCGTGGAGAAGGCTCCTA  R: ATACAGCTGGCCCATGGTAG  F: GAGCCGAGAGTGGGGCTTTGC  R: GCCCTGGCAGGATACCACGC  F: TGTCAAGCTCATTTCCTGGT  R: TAGGGCCTCTCTTGCTCAGT  F: GACCTTCGACAAGGTGTACGTG  R: AGCATCACCGACAGGCTCTTGT  F: CCTGAGACTCAAGCAGAAATGG  R: AGAAGGAAGGTCGGCTTCAGT  F: CAGCGAGGATAAGGTCCAGTAC  R: GCTCACAGTCTTCATTCACAGCC  F: AGGCTTCTGGGCCTTATGTG  R: TGCTTCTCTCGCCAGGAATAC  F: GACATGCCTCTCTCCTGTAGTC  R: GGTGAAGCACATCACTGGTCTC  F: CCCAAAGTTCGTCCCTAAGGTTG  R: ATCAACGCCTGTCCATCCAGCA  F: CCTCTGCTTTCTAGTGATGCCG  R: CGTAAACACGGTCTTGCTCCTG  F: TCTCAGAAACATCCAAGGCC  R: TTTGAGCTGAAGGACTCCATG  F: TCGGAAACCAGCCTCTCAGTGT  R: CTTCTGGATGCAAATGGTCCTTC  F: CGGGTTTCAACGCCGACTA  R: TTGGCACTAGAGACGGACAGA  F: TCATCCGTGTGAGACTGGCTTC  R: GCAGACAGTATCCTGAGTAGGTG  F: TAACCGACGGATGCACTTCTCC  R: CTCCACAGGATAGTTGGGTCTG  F: TCGCTCAGGGTCACAAGAAA  R: CATCAGAGGCAAGGAGGAAAAC  F: CCCAAAGTTCGTCCCTAAGGTTG  R: ATCAACGCCTGTCCATCCAGCA  F: GCCAGGAATTGCTGAGGCACTT  R: GTCTGCGTCAAGACTGCTACAC |
